# Controlled Growth of Graphene‐Skinned Al_2_O_3_ Powders by Fluidized Bed‐Chemical Vapor Deposition for Heat Dissipation

**DOI:** 10.1002/advs.202503388

**Published:** 2025-07-29

**Authors:** Yuzhu Wu, Zhifeng Sun, Ningning Liu, Zhong Wang, Yueming Hu, Tianqi Bai, Tao Wang, Jingyang Chen, Xiaopan Qiu, Xudong Zhang, Fushun Liang, Dongcheng Jiao, Dan Li, Lishuo Han, Wenhu Wang, Qin Xie, Ronghua Zhang, Ali Cai, Yuqi Xia, Haonan Zhai, Zhong‐zhen Yu, Yue Qi, Chu Wang, Peng Gao, Xiucai Sun, Bingyang Cao, Yuqing Song, Zhongfan Liu

**Affiliations:** ^1^ Center for Nanochemistry Beijing Science and Engineering Center for Nanocarbons Beijing National Laboratory for Molecular Science College of Chemistry and Molecular Engineering Peking University Beijing 100871 P. R. China; ^2^ Beijing Graphene Institute (BGI) Beijing 100095 P. R. China; ^3^ State Key Laboratory of Organic‐Inorganic Composites College of Materials Science and Engineering Beijing University of Chemical Technology Beijing 100029 P. R. China; ^4^ School of Aerospace Engineering Tsinghua University Beijing 100084 P. R. China; ^5^ Advanced Materials Institute Qilu University of Technology (Shandong Academy of Sciences) Jinan 250100 P. R. China; ^6^ Academy for Advanced Interdisciplinary Studies Peking University Beijing 100871 P. R. China; ^7^ Electron Microscopy Laboratory School of Physics Peking University Beijing 100871 P. R. China; ^8^ Key Laboratory of Cryogenic Science and Technology Technical Institute of Physics and Chemistry Chinese Academy of Sciences Beijing 100190 P. R. China; ^9^ School of Future Technology University of Chinese Academy of Sciences Beijing 100049 P. R. China; ^10^ State Key Laboratory of Heavy Oil Processing College of Science China University of Petroleum Beijing 102249 P. R. China; ^11^ Key Laboratory of Ocean Energy Utilization and Energy Conservation of Ministry of Education School of Energy and Power Engineering Dalian University of Technology Dalian 116024 P. R. China; ^12^ International Center for Quantum Materials School of Physics Peking University Beijing 100871 P. R. China; ^13^ Collaborative Innovation Centre of Quantum Matter Beijing 100871 P. R. China

**Keywords:** fluidized bed‐chemical vapor deposition, graphene‐skinned Al_2_O_3_, heat dissipation, thermal interface material

## Abstract

The growing demand for high‐performance chips, driven by digitalization and intelligence advancements, is accompanied by rising power consumption, highlighting the critical need for efficient thermal management in electronics. Graphene and its composites, characterized by their exceptional thermal conductivity, hold a distinctive position in this domain. The controlled synthesis of high‐quality multilayer graphene composites, however, remains a significant challenge, hindering the full utilization of graphene's exceptional thermal conductivity. In this research, we present a breakthrough synthesis strategy for graphene‐skinned alumina (Al_2_O_3_) composites via fluidized bed‐chemical vapor deposition (FB‐CVD), constructing a continuous graphene skin with high crystallinity with superior reproducibility between batches. This unique structure enhances thermal conductivity and overall performance by leveraging graphene's superior surface characteristics, interlayer thermal properties, and strong phonon coupling with Al_2_O_3_. The heat flow within the graphene skin surpasses that within the Al_2_O_3_ powders by more than an order of magnitude, establishing a comprehensive heat transfer network in the composite system. The derived thermal interface materials achieve an exceptional thermal conductivity of 6.44 W·m^−1^·K^−1^ and reduce hotspot temperatures in micro‐LEDs by 17.7 °C. This research established a scalable platform for the synthesis of graphene‐skinned ceramic composites, representing a paradigm shift in thermal management strategies for next‐generation nanoelectronics.

## Graphical abstract

This research introduces a facile and scalable FB‐CVD strategy for synthesizing high‐crystallinity, multilayer Gr‐skinned Al_2_O_3_ powders, specifically designed for advanced thermal management applications. The continuous graphene skin establishes a comprehensive heat transfer network, ensuring efficient heat dissipation. The Gr‐skinned Al_2_O_3_ powder‐based TIM demonstrates superior thermal performance with a thermal conductivity of 6.44 W·m^−1^·K^−1^, reducing 17.7°C of the LED.

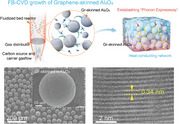

## 1 Introduction

Thanks to the rapid advancements in artificial intelligence (AI) and machine learning, as well as the emergence of 5G and 6G communication technologies, quantum computing, and other cutting‐edge innovations, electronic devices are showing a significant trend of enhanced performance, enhanced intelligence, and seamless integration of functionalities.^[^ ^1^, ^2^, ^3^, ^4^ ^]^ The ever‐increasing computing demands of the digital era necessitate continuous advancements in chip performance and density, while simultaneously intensifying the significance of heat dissipation challenges.^[^ ^5^, ^6^, ^7^ ^]^ The potential solutions for improving heat dissipation include the utilization of new materials, liquid cooling technology, and more efficient heat dissipation designs.^[^ ^8^, ^9^, ^10^, ^11^, ^12^ ^]^ However, it is important to note that these solutions are rooted in the preparation of suitable materials. Materials possessing ultra‐high thermal conductivity (*k*) are of technological significance and practical breakthroughs.^[^ ^13^ ^]^

The thermal interface materials (TIMs) are crucial functional components for effectively managing heat dissipation in high heat flux devices, and they play a vital role in reducing the thermal resistance between integrated circuit (IC) chips and heat sinks in electronic devices due to their straightforward preparation process and absence of additional energy consumption.^[^ ^10^, ^14^, ^15^, ^16^ ^]^ The composition of TIMs typically consists of polymers and highly thermal conductive fillers, such as metallic oxides (Al_2_O_3_, etc.), boron nitride (BN), aluminum nitride (AlN), and carbon‐based materials.^[^ ^5^, ^17^, ^18^ ^]^ These fillers enable the construction of a 3D thermal conductivity network. The intrinsic thermal conductivity of the polymer matrix is typically low under normal circumstances, thus making the filler the primary determinant of thermal conductivity in TIMs. Spherical α‐Al_2_O_3_ can achieve a uniform dispersion within the polymer matrix, thus becoming the most commonly utilized thermal conductive filler, owing to its superior sphericity, diminished surface energy, enhanced surface fluidity, and affordable price. The thermal conductivity of α‐Al_2_O_3_ powder (*k*≈30 W·m^−1^·K^−1^) is limited, rendering it insufficient for addressing modern heat dissipation challenges. This limitation has spurred interest in 2D carbon materials, particularly graphene, which demonstrates exceptional in‐plane thermal conductivity (*k*≈3000–5000 W·m^−1^·K^−1^).^[^ ^13^ ^]^ Such superior thermal transport capability, primarily mediated by efficient phonon propagation, positions graphene as a transformative solution for next‐generation thermal management systems.^[^ ^13^, ^19^, ^20^ ^]^ The material's thermal superiority stems from its unique phonon transport mechanism, which enables effective thermal energy transfer across filler‐matrix interfaces. However, practical implementation faces two critical challenges. First, the requirement for high‐quality crystalline structures in phonon‐dominant thermal radiation systems imposes stringent demands on graphene synthesis techniques.^[^ ^21^, ^22^ ^]^ Second, inherent material limitations, including the stacking tendency of graphene/graphite layers and inconsistent quality of commercial graphene products, substantially compromise the full utilization of their theoretical thermal properties. Previous research has found that the best thermal conductivity of the composite containing multilayer graphene films as fillers was 5.1 W·m‐1·K^−1^ when the fraction reached 10 vol%.^[^ ^23^ ^]^ When the fraction exceeds 10 vol%, the viscosity escalates exponentially, disrupting filler dispersion homogeneity and amplifying surface roughness. This affects the interfacial contact resistance of TIMs and the composite's overall performance. The existing methods fail to address the challenges associated with improving graphene quality, preventing agglomeration, overcoming limited filling capacity, resolving deposition problems, and establishing a stable network for enhancing thermal conductivity.

**Figure 1:**
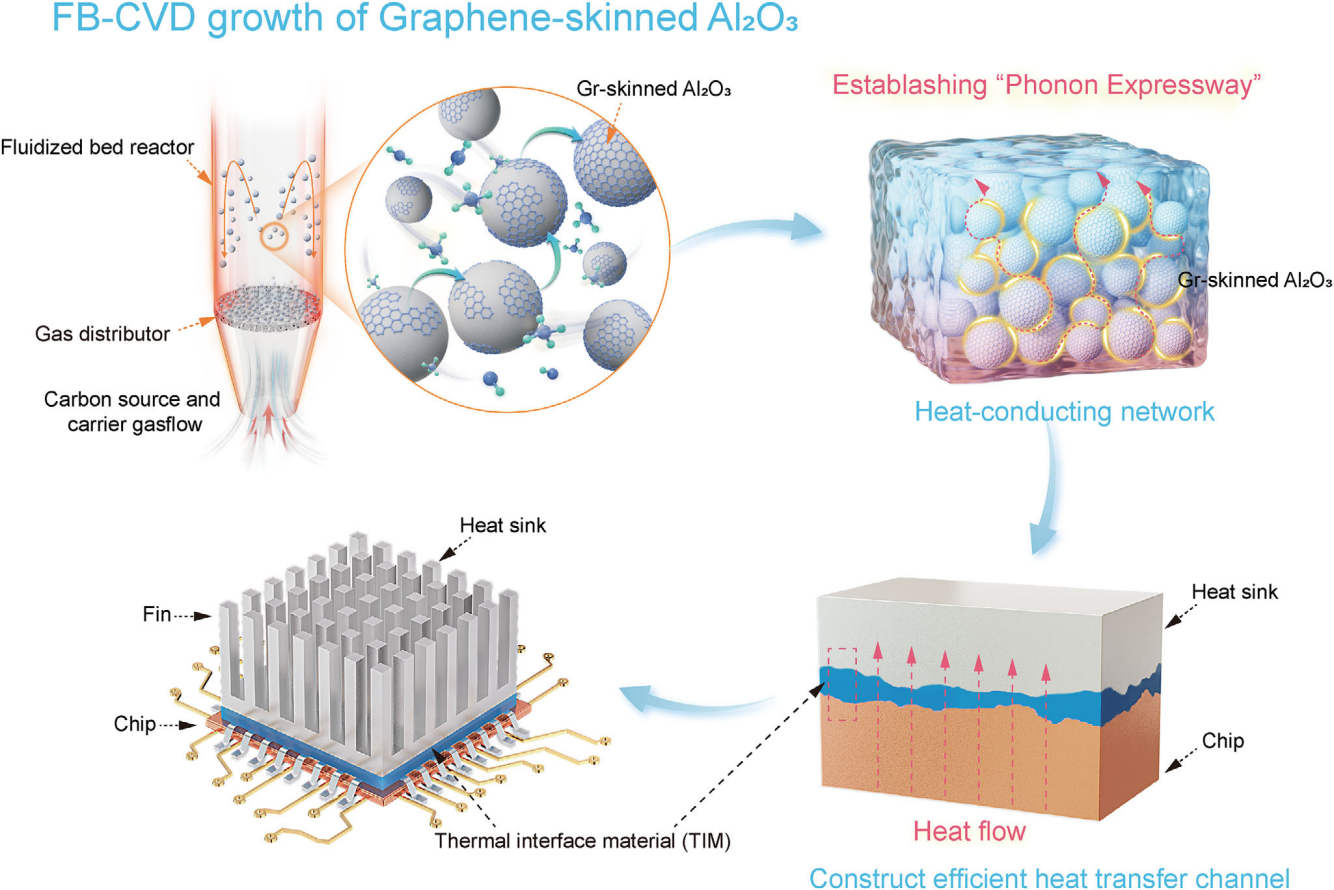
Schematic illustration for the preparation process of Gr‐skinned Al_2_O_3_ powder‐based TIM. A gas mixture comprising CH_4_ as the carbon precursor and carrier gas is supplied in an upflow manner to ensure a continuous provision of carbon atoms and sufficient fluidization, while Al_2_O_3_ powders are positioned on the gas distributor. The obtained Gr‐skinned Al_2_O_3_ powders were filled into the polymer matrix to establish a “Phonon Expressway” for rapid heat conduction. Gr‐skinned Al_2_O_3_ based TIM is capable of filling the micro gap that occurs when the IC chip and heat sink are engaged, thereby efficiently conducting the heat generated by the chip to the heat sink due to the continuous graphene skin as a heat transfer channel.

Herein, we report the rational design and scalable synthesis of “Graphene‐skinned Al_2_O_3_ powder composites” through an advanced fluidized bed‐chemical vapor deposition (FB‐CVD) technique. This innovative approach enables the direct growth of continuous multilayered graphene skins on Al_2_O_3_ particulate substrates, establishing a novel class of thermally conductive fillers for enhanced heat dissipation applications (**Figure** **1**).^[^ ^24^ ^]^ The architecture addresses two critical challenges in conventional composite systems: i) preserving the established processing compatibility of industrial alumina powders while ii) effectively mitigating graphene sheet restacking and phase segregation phenomena within polymer matrices. Compared to traditional CVD methodologies, the FB‐CVD process demonstrates superior advantages in three key aspects: i) enhanced crystalline quality of deposited graphene layers, ii) improved thermal transport efficiency through optimized gas‐solid interactions, and iii) scalable production capability for industrial‐grade powder quantities.^[^ ^25^ ^]^ The aforementioned advantages enable precise control over the quality and layer number of graphene skin, thereby facilitating stable mass production of Gr‐skinned Al_2_O_3_ powder. Additionally, we incorporated machine learning techniques into our investigation of the thermal conductivity enhancement in Gr‐skinned Al_2_O_3_ powders. Our findings have revealed that the exceptional thermal conductivity of graphene skin in terms of both in‐plane transport and interlayer transport between graphene layers significantly contributed to this improvement. Critically, this enhancement was achieved by preserving standard TIM fabrication protocols without requiring process modifications, enabling direct substitution while leveraging graphene's thermal transport and seamless integration into existing electronic packaging workflows. The contact between the stacked powders of the graphene skin establishes an unobstructed “Phonon Expressway” that facilitates efficient heat transfer in all directions. The exceptional thermal conductivity of the large‐area continuous graphene skin results in a significantly higher heat flux compared to that of the Al_2_O_3_ substrate, with predominant heat transfer occurring along the graphene skin. A layer of Gr‐skinned Al_2_O_3_ based TIM is sandwiched between the 50 W light‐emitting diode (LED) lamp and the heat sink, which exhibits superior thermal dissipation performance and reduces the device temperature by 17.7°C. This performance enhancement directly translates to enhanced operational reliability and reduced thermal fatigue failure in high‐power electronic devices.

## 2 Results and Discussion

Herein, we present an optimized graphene growth strategy leveraging the well‐characterized surface properties of α‐Al_2_O_3_ substrates, which have been extensively studied as model templates for epitaxial growth of metallic, 2D, and semiconductor architectures.^[^ ^26^, ^27^ ^]^ Cross‐sectional transmission electron microscopy (TEM) analysis (Figure S1, Supporting Information) confirms the atomically smooth terraces of focused ion beam (FIB)‐processed α‐Al_2_O_3_ particulates, providing an ideal platform for conformal graphene deposition. Following a thorough investigation, we opted for the high‐temperature FB‐CVD growth technique over the conventional CVD approach. This decision was driven by the fact that the superior thermal transport characteristics of fluidized particulate systems compared to gaseous media, achieving 5–10 enhanced heat transfer efficiency.^[^ ^28^, ^29^, ^30^, ^31^ ^]^ Additionally, dynamic gas‐solid interactions enable uniform thermal transport across industrial‐scale powder quantities and precise control over graphene layer stacking through modulated fluidization parameters (Figure 1). The graphene deposition process was implemented in a custom‐designed fluidized bed reactor (Figure S2, Supporting Information) employing CH_4_/H_2_/Ar precursor mixtures under optimized upflow conditions. During the growth stage, a gas mixture comprising CH_4_ as the carbon source and carrier gas (H_2_ and Ar) was introduced in an upflow manner to ensure a continuous supply of carbon atoms while maintaining full fluidization. When the carbon source and carrier gas flow are activated, the solid powders become suspended by the upward airflow, resulting in a fluid with enhanced gas‐solid contact. The growth was performed at 1100 °C, yielding 150–200 g per batch depending on particle density and size, with the objective of achieving homogeneous coverage of continuous graphene film on the target substrate. A multiscale investigation framework combining cold‐flow modeling and computational fluid dynamics (CFD) simulations systematically elucidated the fluidization dynamics across particle size distributions (40, 70 µm). CFD simulations clarified the key role of gas flow in the growth of graphene in a fluidized bed reactor. An appropriate gas flow can effectively disperse particle agglomerates in localized regions to enhance the microscale diffusion of heat and particulate matter to achieve powder spatial homogeneity. This computational‐experimental synergy enabled predictive tuning of growth parameters (Figures S3 and S4, Table S1, and Videos S1–S4, Supporting Information).

Building upon the precisely controlled graphene growth enabled by our multiscale fluidization strategy (5, 40, 70 µm Al_2_O_3_), we implement a poly‐disperse filler architecture incorporating 5, 40, and 70 µm particles. This hierarchical gradation allows the interstitial voids between larger particles to be systematically occupied by smaller counterparts, achieving packing densities exceeding 95.3%, which is an obvious improvement over mono‐disperse systems. The thermal conductivity of TIMs is intricately linked to the distribution of thermal conductive fillers. With identical filler content, an optimized filler gradation facilitates a denser packing of the filler within the polymer matrix, thus enhancing inter‐filler contact and forming additional thermal conduction pathways. This, in turn, improves the phonon transfer efficiency within the material and enhances the thermal conductivity of the composite.

**Figure 2:**
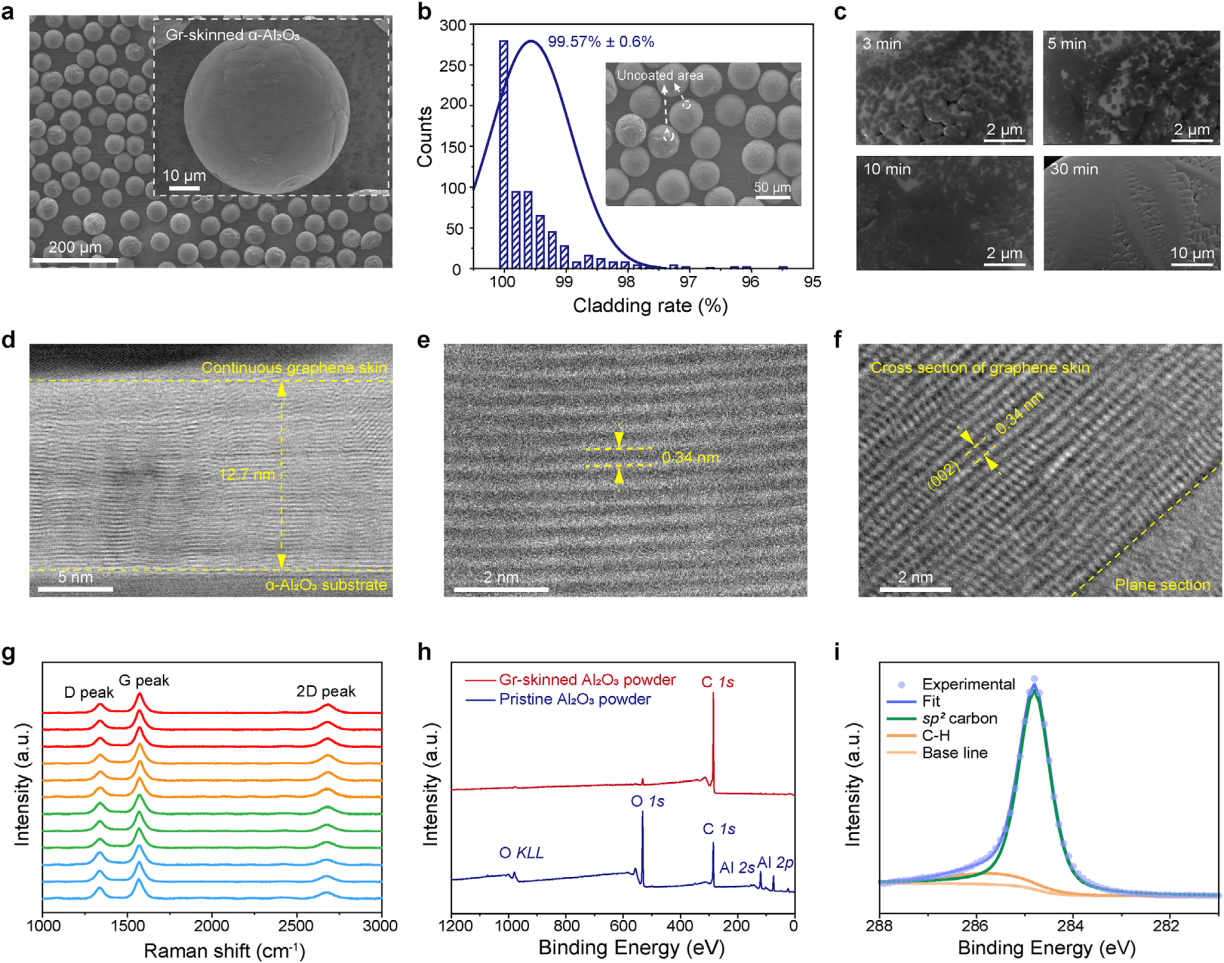
Characterization of Gr‐skinned Al_2_O_3_ powder materials. a) SEM image of the Gr‐skinned α‐Al_2_O_3_ powders after 30 min FBCVD growth, while the uncoated area shows a lighter contrast difference. Inset: Single Gr‐skinned Al_2_O_3_ powder, fully covered by continuous graphene skin. b) Cladding rate characterization of Gr‐skinned Al_2_O_3_ powder. Inset: SEM image of 40 µm Gr‐skinned Al_2_O_3_ powder. c) SEM images of Gr‐skinned Al_2_O_3_ powder obtained with a growth time of 3–30 min. d) Low‐magnification cross‐sectional STEM image of continuous graphene skin and α‐Al_2_O_3_ substrate. e) Cross‐sectional high‐magnification STEM image of continuous graphene skin. f) Atomically resolved TEM image of transferred continuous graphene skin. g) Raman spectra of Gr‐skinned α‐Al_2_O_3_ powders with good uniformity. h) Full‐range XPS spectra of pristine Al_2_O_3_ powder and Gr‐skinned Al_2_O_3_ powder. i) C*1s* XPS spectra of Gr‐skinned α‐Al_2_O_3_ powder.

Laboratory‐scale graphene growth was conducted using 150 g of Al_2_O_3_ powder under optimized FB‐CVD conditions (1100°C, atmospheric pressure, Ar/H_2_/CH_4_ = 900/300/300 sccm, 30 min). Take 40 µm Al_2_O_3_ powders for example, Figures S5 and S6 (Supporting Information) present photographic and scanning electron microscopy (SEM) images of both the pristine Al_2_O_3_ powders and the Gr‐skinned Al_2_O_3_ powders. It is noteworthy that, following the growth of graphene, the powder exhibits a grey color while retaining its spherical morphology. The uniform contrasts in the SEM confirmed the continuous full coverage of graphene skin on Al_2_O_3_ powders (**Figure** **2**a) while preserving its original spherical morphology. The inset image illustrates a singular Gr‐skinned Al_2_O_3_ powder that is entirely encapsulated by graphene skin. To investigate the cladding rate, we devised a robust methodology grounded in SEM image analysis. Areas covered by graphene exhibit a darker contrast, whereas uncovered regions devoid of graphene coverage appear as white spots. The uncovered region in the SEM image was isolated and rectified using Photoshop. Subsequently, the corrected area was quantified to determine the cladding ratio post‐transformation. Quantitative assessment via SEM image analysis revealed an exceptional graphene coverage efficiency of 99.57 ± 0.6% for 40 µm Gr‐skinned Al_2_O_3_ powders, demonstrating excellent uniformity (Figure 2b, Figure S7, Supporting Information). This phenomenon further corroborates that within the fluidized bed, the gas phase and the powder are in intimate contact. The Al_2_O_3_ powder circulates due to the influence of the gas phase, which facilitates fast and uniform deposition of graphene. The SEM image presented in Figure 2c demonstrates that a significant number of graphene nuclei have formed on the Al_2_O_3_ powder within ≈3 min, indicating the much higher mass and heat transfer efficiency and shorter incubation time required for graphene nucleation in the FB‐CVD process. As the growth duration increases, these graphene nuclei coalesce to form a continuous graphene layer on the substrate.

Precise quantification of graphene skin thickness was achieved through the FIB‐TEM characterization approach. The cross‐sectional morphology of the Gr‐skinned Al_2_O_3_ powder in Figure 2d was obtained using FIB technology and subsequently characterized via spherical aberration corrected‐transmission electron microscopy (AC‐TEM), revealing a thickness of ≈12.9 nm, which corresponds to roughly 38 layers of graphene with the layer in conformal contact with the substrate (1100°C, atmospheric pressure, Ar/H_2_/CH_4_ = 900/300/300 sccm, 35 min). High‐resolution STEM imaging confirmed the crystalline integrity of the graphene layers, showing a characteristic 0.34 nm interlayer spacing (Figure 2e), which matches theoretical graphene lattice parameters. Besides, precise modulation of graphene coating thickness was achieved by temporal control of the growth process under standardized conditions (1100°C, atmospheric pressure, Ar/H_2_/CH_4_ = 900/300/300 sccm). As systematically demonstrated in Figure S8 (Supporting Information), high‐resolution transmission electron microscopy (HR‐TEM) analysis reveals the correlation between growth duration and graphene thickness. When altering the growth time from 10, 20, 30, 60, 120 min, the thicknesses of the corresponding graphene skin are 4.0, 5.0, 10.1, 25.1, 40.0 nm, equivalent to 12, 15, 30, 75, 120 graphene layers. The energy dispersive spectrometer (EDS) elemental mapping of the 30‐min sample (Figure S9, Supporting Information) confirms the conformality, with a sharp C/Al interface transition zone, consistent with the measured 10.1 nm graphene thickness. To further validate the structural continuity, hydrochloric acid (HCl)‐etched samples (72‐h, see more details in Methods) were analyzed via HR‐TEM (Figure 2f), revealing collapsed graphene stacks maintaining 0.34 nm interlayer distances. To probe the defect density and structural integrity of graphene skin, we strategically analyzed partially etched specimens (24‐h HCl treatment) retaining residual Al_2_O_3_ cores. This protocol enables simultaneous evaluation of graphene‐substrate interfacial, coating continuity, and uniformity across curved surfaces. High‐resolution TEM of the graphene‐Al_2_O_3_ interface and EDS map scans (Figure S10, Supporting Information) reveal an intimate graphene‐Al_2_O_3_ contact, confirming conformality. The proportion of graphene by mass is then estimated by thermogravimetric analysis (TGA) analysis (≈0.15‐1.53%), which can further confirm the increase of graphene layers with the elevating growth time. As illustrated in the differential thermogravimetric analysis (DTG) graphs, the temperature of maximum mass change rate (T_max_) can be attributed to the maximum external heat energy required to overcome the bonding within the carbon lattice structure, which increased significantly (712–804°C) with the elevation of graphene layers (Figure S11, Supporting Information). The enhanced thermal stability (T_max_ elevation) with increasing graphene layers correlates with improved crystallinity, manifested by reduced defect density. Such crystallographic perfection minimizes oxidative attack initiation sites while strengthening in‐plane covalent bonding, requiring higher energy to rupture C─C bonds. Synergistically, multilayer stacking further impedes oxygen permeation through tortuous interlayer pathways, collectively elevating the thermal degradation threshold. To correlate structural integrity with growth conditions, Raman spectroscopy provides critical metrics for defect density and thickness evaluation. The D‐peak intensity in the Raman spectrum is observed at ≈1350 cm^−1^ and is commonly associated with the vibration peak induced by disorder in graphene, resulting from lattice movements distant from the center of the Brillouin zone.^[^ ^32^ ^]^

The I_D_/I_G_ (the ratio of the D‐peak to G‐peak intensity) is a crucial metric for assessing the defect density within the graphene layer and serves as a reliable criterion for evaluating the quality of Gr‐skinned Al_2_O_3_ powder, enabling precise tracking of graphene quality evolution during process optimization (Figure S12, Supporting Information). Figure 2g was acquired from a random selection of 12 Gr‐skinned Al_2_O_3_ powders, with the data normalized to the G‐peak intensity of each individual spectrum. The analysis revealed consistent I_D_/I_G_ and I_2D_/I_G_ (the ratio of the 2D‐peak to G‐peak intensity), as well as a uniform peak shape, which further indicates the homogeneity of the graphene skin thickness and crystalline quality (Table S2, Supporting Information). These findings confirm the high stability and reliability of our production process. As illustrated in Figure S13 (Supporting Information), the defect density (I_D_/I_G_) follows a trend that the defect density in graphene first diminishes and then rises with the elevating C/H ratio, attaining a minimum I_D_/I_G_ (0.55) value at a C/H ratio of 1:1. The analysis revealed a progressive decline in Raman I_D_/I_G_ ratio with increasing growth duration, demonstrating a progressive stabilization trend beyond 30 min. This temporal evolution reflects defect healing through hydrogen‐mediated edge reconstruction and nascent defect formation from prolonged carbon supersaturation. The observed plateauing of defect density after 30 min is consistent with thermodynamic equilibrium between precursor decomposition and graphene crystallization rates. Through systematic parameter analysis, the optimal growth condition was identified as 1100 °C during 30 min under atmospheric pressure with Ar/H_2_/CH_4_ flow rates of 900/300/300 sccm, considering crystallinity and energy consumption. To validate the batch‐to‐batch reproducibility critical for industrial scaling, five independent production batches (40 µm Al_2_O_3_) were processed under identical FB‐CVD conditions (1100 °C, atmospheric pressure, Ar/H_2_/CH_4_ = 900/300/300 sccm, 30 min). For each batch, 10 particles were randomly sampled for statistical characterization, with inter‐batch variability quantified through coefficient of variation (CV) analysis. Figure S14 (Supporting Information) presents representative Raman spectra of Gr‐skinned Al_2_O_3_ powders across five production batches, exhibiting consistent D, G, and 2D peak profiles. Quantitative analysis reveals minimal variability in crystallinity (I_D_/I_G_ = 0.56 ± 0.03, CV = 0.050) and layer thickness (I_2D_/I_G_ = 0.37 ± 0.01, CV = 0.037) between batches of 50 sampled powders, confirming the process robustness. Intra‐batch uniformity was further demonstrated by <9% variation in both I_D_/I_G_ and I_2D_/I_G_ (Figure S14c,e, Supporting Information), meeting industrial‐grade repeatability standards. The achieved CV values (<0.09) position this method as a viable pathway toward mass production of Gr‐skinned Al_2_O_3_ powder materials. Our FB‐CVD process exhibits exceptional batch‐to‐batch reproducibility, which stands out as a critical prerequisite for industrial adoption. To comprehensively link process consistency with atomic‐scale material quality, we further probed the chemical bonding and crystallographic features of Gr‐skinned Al_2_O_3_ powder. X‐ray photoelectron spectroscopy (XPS) was employed to ascertain the binding energies of electrons, which facilitated the identification of the chemical characteristics and compositional makeup of the graphene skin surface.^[^ ^27^ ^]^ The full spectra, which illustrate the contrast between pristine Al_2_O_3_ and Gr‐skinned Al_2_O_3_ powder, can be found in Figure 2h. Figure 2i presents the C*1s* XPS spectrum of Gr‐skinned Al_2_O_3_ powder, illustrating the characteristic signals of graphene with a predominant *sp^2^* carbon peak (≈284.80 eV) and a weak C─H peak (≈285.70 eV). The dominant presence of *sp^2^* carbon is speculated to be the result of the high mass and heat transfer, consequently forming hexatomic rings. X‐ray diffraction (XRD) patterns of pristine α‐Al_2_O_3_ and Gr‐skinned Al_2_O_3_ powder at ≈1100°C of different growth times were demonstrated in Figure S15 (Supporting Information).

These analyses collectively confirm the formation of multi‐layer, homogeneous graphene skin in conformal contact with the Al_2_O_3_ substrate, which stands out as a structural prerequisite for establishing uninterrupted thermal transport pathways. To unravel how this atomic‐scale architecture governs macroscopic heat dissipation, we bridge experimental observations with theoretical simulations that decode interfacial heat transfer mechanisms.

**Figure 3:**
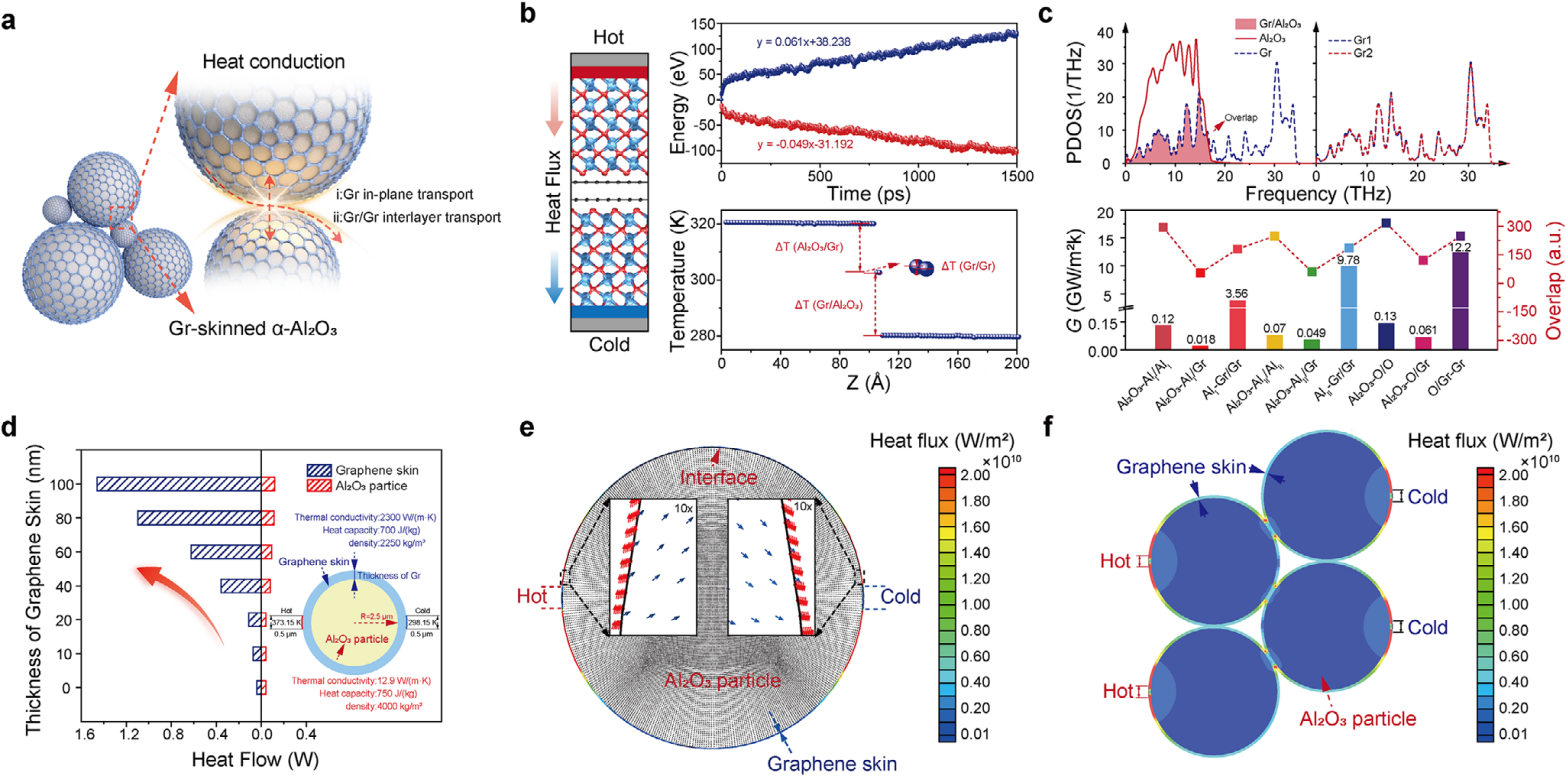
Thermal conductivity of Gr‐skinned α‐Al_2_O_3_ powders. a) Schematic illustration of interfacial heat conduction between Gr‐skinned α‐Al_2_O_3_ powders. b) Interface modelling of Gr‐skinned Al_2_O_3_ with C‐O as the contact surface (left) in NEP simulation, along with its accumulated energy (heat sink, heat source) and temperature profile (right). c) PDOS of typical O terminated Al_2_O_3_/Gr (abbreviated as Al_2_O_3_‐O/Gr) and Gr/Gr interfaces in Gr‐skinned Al_2_O_3_ (top) as well as thermal conductivity values and their overlap at various contact interfaces before and after Gr‐skinned Al_2_O_3_ (bottom). d) Comparison of heat flow between graphene skin with different thicknesses and Al_2_O_3_ powder. e) Heat flux vector of Gr‐skinned α‐Al_2_O_3_ powder. f) Heat‐flux distribution of stacked Gr‐skinned α‐Al_2_O_3_ powders.

The conformal graphene skin observed on Al_2_O_3_ powder (**Figure** **3**a) suggests a continuous pathway for phonon‐mediated thermal transport. We employed multiscale simulations combining non‐equilibrium molecular dynamics (NEMD)^[^ ^33^ ^]^ and machine learning potentials (MLPs). Recent advances in MLPs have facilitated large‐scale thermal conductivity simulations, achieving computational accuracy that rivals quantum‐level computations. Our simulations compared the thermal characteristics of both pure‐phase Al_2_O_3_ and Gr‐skinned Al_2_O_3_ materials, with the detailed force field development process and accuracy verification provided in Figures S16–S18 and Table S3 (Supporting Information).^[^ ^34^, ^35^, ^36^, ^37^, ^38^, ^39^, ^40^, ^41^ ^]^

First, the thermal conductivity (*k*) of pure‐phase Al_2_O_3_ can be obtained using the Fourier's law calculation:^[^ ^42^, ^43^, ^44^ ^]^

(1) $$k=-\frac{J}{\nabla T}$$

where *J* is the heat flux along the z‐axis, and ▽*T* represents the temperature gradient. The heat flux can be determined by the energy transfer rate:

(2) $$J=\frac{\mathrm{dE}/\mathrm{dt}}{S}\;$$

where *E* denotes accumulated energy, *S* is the cross‐sectional area of the computational model. Based on the cumulative energy and temperature gradient output from the NEMD simulations (Figure S17, Supporting Information), the thermal conductivity of the infinitely large Al_2_O_3_ bulk calculated using the machine learning trained neuroevolution machine learning potentials (NEP) is 40.16 W·m^−1^·K^−1^, which is very close to the experimental results reported by Vera‐Londono L and Paterson J.^[^ ^35^, ^38^ ^]^ Subsequently, we constructed Gr‐skinned Al_2_O_3_ interfacial structures represented in Figure 3b, consisting of two oxygen‐terminated Al_2_O_3_ (001) planes stacked with graphene (referred to as Al_2_O_3_‐O/Gr), as well as a purely Al_2_O_3_ stacked Al_2_O_3_‐O/O structure. Both models were set to a size of 25 Å × 29 Å × 200 Å for comparative calculations. The thermal properties of Gr‐skinned Al_2_O_3_ powders are listed in Table S4 (Supporting Information). In the Gr‐skinned Al_2_O_3_ system, the heat transfer between Gr/Al_2_O_3_ structural units can be divided into three main processes: (I) heat transfer across the Al_2_O_3_‐O/Gr interface (Al_2_O_3_‐O/Gr), (II) heat transfer between graphene layers (O‐Gr/Gr), and (III) heat transfer at the graphene and Al_2_O_3_ interface (Gr/O‐Al_2_O_3_). Processes I and III are considered reversible and equivalent (to avoid redundancy, the following discussion will focus only on processes I and II). We established the heat flow direction based on the heat source and heat sink as shown in Figure 3b and used interfacial thermal conductance (*G*) to describe the heat transfer capability between the materials on both sides of the interface. The calculation formula for interfacial thermal conductance is as follows:^[^ ^42^ ^]^

(3) $$G=\frac{q}{A\Delta T}\;\;$$

where *q* is the heat flow, *A* denotes the interfacial cross‐sectional area, and Δ*T* is the temperature difference.

By utilizing the thermal conductivity parameters output from the NEMD simulations with a trained NEP (the right side of Figure 3b), the interfacial thermal conductance for the I and II processes was calculated separately. The computational results in Figure 3c indicate that the interfacial thermal conductance at the Al_2_O_3_‐O/O interface is 0.13 GW·m^−2^·K^−1^, while the interfacial thermal conductance of the Al_2_O_3_‐O/Gr interface in the Gr‐skinned Al_2_O_3_ system is 0.061 GW·m^−2^·K^−1^, and the interlayer conductance of the O‐Gr/Gr interface is 12.2 GW·m^−2^·K^−1^. Among these, the Al_2_O_3_‐O/Gr interface exhibits the lowest interfacial thermal conductance, while the O‐Gr/Gr interface is the highest. The interlayer thermal conductance of O‐Gr/Gr is two orders of magnitude higher than the interfacial thermal conductance of Al_2_O_3_‐O/O before introducing graphene. This can be attributed to the identical interfacial structures and atomic arrangements of O‐Gr/Gr and Al_2_O_3_‐O/O, which allows phonons to pass through the interface more easily compared to heterogeneous interfaces, thereby reducing the scattering at the interface. Consequently, the heat transfer is more efficient through phonon propagation, leading to higher interfacial thermal conductance.^[^ ^45^ ^]^ Additionally, O‐Gr/Gr possesses stronger van der Waals forces compared to Al_2_O_3_‐O/O, further enhancing the thermal transport efficiency relative to Al_2_O_3_‐O/O. The computational results for interfacial models based on other crystal planes (including Al_2_O_3_‐Al_I_/Al_I_, Al_2_O_3_‐Al_II_/Al_II_, Al_2_O_3_‐Al_I_/Gr, Al_2_O_3_‐Al_II_/Gr, Al_I_‐Gr/Gr, and Al_II_‐Gr/Gr shown in Figure S19, Supporting Information) indicate that the interlayer thermal conductance of the homogeneous Gr/Gr interface is generally higher than that of other heterogeneous interfaces. Furthermore, the Al_2_O_3_‐O/Gr interface exhibits the highest interfacial thermal conductance during the process of limiting heat transport between Al_2_O_3_ and graphene. This can be attributed to the higher electron density concentrated on the oxygen atoms in Al_2_O_3_, which gives the oxygen atoms greater electronegativity. As a result, the stronger bonding between Al_2_O_3_ and graphene slightly enhances the heat transport capacity at the interface.^[^ ^46^ ^]^

The differences in thermal conductivity across disparate interfaces can also be ascribed to the different interfacial phonon density of states (PDOS). The calculation formula for PDOS is as follows:^[^ ^47^, ^48^ ^]^

(4) $$\mathrm{PDOS}\left(w\right)=\int_{-\infty }^{\infty }e^{-\mathrm{iwt}}\mathrm{VACF}\left(t\right)\mathrm{dt}\;\;$$

where,

(5) $$\mathrm{VACF}\left(t\right)=\frac{1}{N}\sum_{j=1}^{N}<v_{j}\left(0\right)v_{j}\left(t\right)>$$

where W is the vibrational frequency of the phonon, N is the number of atoms, *v*_j_(t) is the velocity vector of the j^th^ atom at moment t, and <…> is the systematic mean. As shown in Figure 3c and Figure S20 (Supporting Information), among all heterogeneous interfaces, the PDOS curve of Al_2_O_3_ in the Al_2_O_3_‐O/Gr interface is noticeably broader. This indicates a relative reduction in phonon scattering and boundary scattering at the interface, as well as an increase in the number of phonons. The PDOS of graphene increases and flattens in both the 0–7 THz (low‐frequency range) and 7–20 THz (mid‐frequency range) ranges, indicating the introduction of a novel anharmonic channel at this interface. The opening of these anharmonic channels triggers nonlinear effects, which promote inelastic scattering between phonons and result in the energy transfer from high‐frequency phonons to low‐frequency phonons, thereby enhancing the interfacial thermal conductance.^[^ ^49^ ^]^ Meanwhile, since low and mid‐frequency phonons dominate the heat transport, the degree of phonon coupling at the Gr‐skinned Al_2_O_3_ interface can be quantified by integrating the overlapping area of interfacial phonons in the low and mid‐frequency range (0–20 THz):^[^ ^48^ ^]^

(6) $$S=\int_{-\infty }^{\infty }\min\left\{P_{X}\left(w\right)P_{Y}\left(w\right)\right\}\mathrm{dw}$$

In the equation, *P*_X_(w) and *P*_Y_(w)) represent the PDOS of the materials on either side of the interface. Calculations show that the phonon coupling degree at the Al_2_O_3_‐O/Gr interface reaches 0.208, the highest among all heterogeneous interfaces. The strong phonon coupling facilitates atomic resonance at the same frequency across the interface, thereby improving the efficiency of thermal transport at the interface. It is noteworthy that compared to heterogeneous interfaces (Al_2_O_3_‐Al/Gr, Al_2_O_3_‐O/Gr), the PDOS coupling degree on both sides of homogeneous interfaces (Al_2_O_3_‐Al/Al, Al_2_O_3_‐O/O, Al‐Gr/Gr, and O‐Gr/Gr) is significantly higher. This is because the homogeneous interfaces have identical structures and atomic arrangements, allowing phonons to pass through the interface more easily without scattering. Additionally, the consistent frequency of homogeneous phonons ensures efficient interfacial heat conduction.^[^ ^45^ ^]^ This implies that the graphene coating introduced during the preparation of Gr‐skinned Al_2_O_3_ composites can establish a rich network for heat transfer within the composite system. In addition to graphene's excellent in‐plane thermal transport capability,^[^ ^50^ ^]^ the heat transfer efficiency between graphene layers can also be well maintained.

To further substantiate the enhanced thermal conductivity of Gr‐skinned Al_2_O_3_ powder, we conducted a comprehensive analysis of its temperature, heat flux, and heat transfer characteristics utilizing the commercial software ANSYS. The material model and boundary conditions are illustrated in Figure S21 (Supporting Information), with Al_2_O_3_ serving as the base powder and graphene skin as the coating. The numerical grids, CFD model, and material properties are detailed using the integrated computer engineering and manufacturing code for computational fluid dynamics (ICEM CFD) and Fluent modules within ANSYS. It can be seen in Figure S22 (Supporting Information) that when the grid size is less than 6 nm, the heat flow tends to stabilize and remain basically unchanged. Therefore, the grid size for subsequent simulation calculations was set at 6 nm in consideration of computational efficiency.

To further analyze the relationship between the thickness of the graphene layer and the heat flow, we established the model with the thickness of the coating varying from 10, 20, 40, 60, 80, and 100 nm. As shown in Figure 3d, the heat flow through the Gr‐skinned Al_2_O_3_ powders rapidly increases with the thickening of the graphene skin. Compared to the Al_2_O_3_ powders without a graphene skin, the total heat flow of the Gr‐skinned Al_2_O_3_ powders is enhanced by 1.78, 2.73, 8.70, 15.15, 26.68, and 35.48 times, respectively. Furthermore, the heat flow passing through the graphene surface layer accounts for 43.67%, 63.25%, 77.92%, 85.36%, 89.71%, and 91.85% of the total heat flow, respectively. This suggests that the primary contribution to the increase in heat flow stems from the graphene skin, with heat predominantly transferred within it.

**Figure 4:**
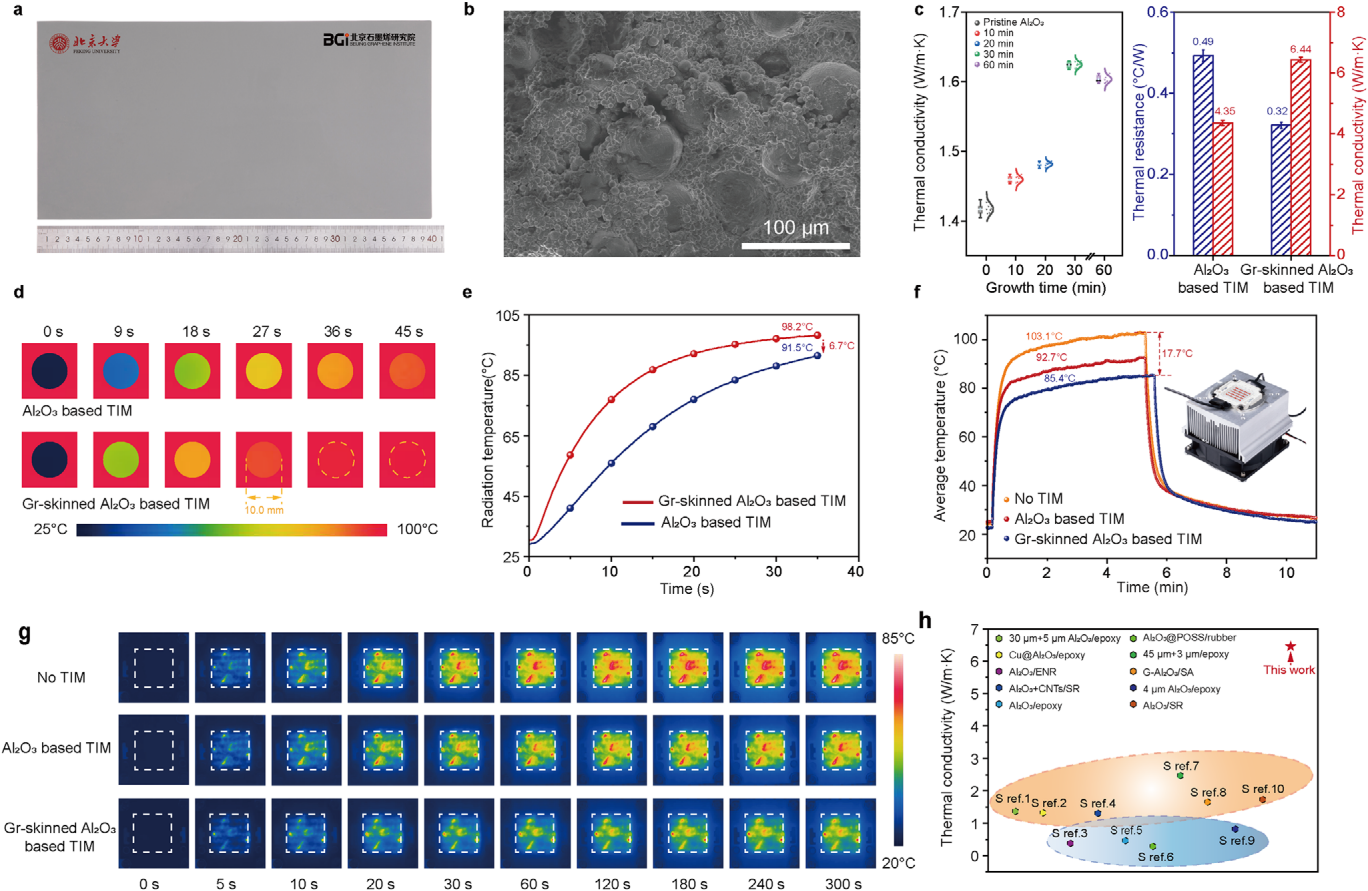
Thermal conductivity of Gr‐skinned Al_2_O_3_ powder‐based TIM. a) Photograph of Gr‐skinned Al_2_O_3_ powder‐based TIM with a size of 20 × 40 cm. b) Cross‐sectional SEM image of Gr‐skinned Al_2_O_3_ powder‐based TIM. c) Thermal conductivity of Gr‐skinned Al_2_O_3_ powder tablets of different growth times along with Al_2_O_3_ powder tablet by Hot Disk analysis (left) and comparison of thermal conductivity and thermal resistance between pristine Al_2_O_3_ powder‐based TIM and Gr‐skinned Al_2_O_3_ powder‐based TIM (right). d) Infrared camera images of the pristine Al_2_O_3_ powder‐based TIM (above) and Gr‐skinned Al_2_O_3_ powder‐based TIM (bottom). e) Surface radiation temperature variation with heating time of pristine Al_2_O_3_ powder‐based TIM and Gr‐skinned Al_2_O_3_ powder‐based TIM. f) Comparison of Al_2_O_3_ powder‐based TIM and Gr‐skinned Al_2_O_3_ powder‐based TIM in devices. g) Temperature cloud diagrams of the LED during the heating process when using no TIM, Al_2_O_3_ based TIM, and Gr‐skinned Al_2_O_3_ based TIM. h) Comparison of thermal conductivity between our work and other TIMs related to the modification of Al_2_O_3_ thermal filler.^[^ ^51^, ^52^, ^53^, ^54^, ^55^, ^56^, ^57^, ^58^, ^59^, ^60^ ^]^

To intuitively visualize heat propagation pathways, we conducted comparative thermal flux analyses using two representative thicknesses (10.1 and 100 nm). Uniform‐length arrows were employed to denote heat transfer directions, while color gradients (blue‐to‐red scale) quantitatively encoded heat flux density magnitudes, with warmer colors corresponding to higher heat flux. This dual‐parameter visualization protocol eliminates ambiguities in interpreting anisotropic thermal transport, particularly critical for substantiating the enhanced thermal conductivity of Gr‐skinned Al_2_O_3_ powder. Figure 3e illustrates the heat flux vector of a Gr‐skinned Al_2_O_3_ powder when subjected to a constant temperature heat source and a cold source (the graphene thickness was set at 10.1 nm, corresponding to the experimentally optimized value). It is evident that, owing to the superior thermal conductivity of graphene, the heat flux within the coating is approximately two orders of magnitude greater than that within the substrate powder. The heat flux vectors within the graphene skin primarily align with the circumferential direction of the skin, indicating the formation of preferential thermal conduction pathways within the graphene skin as a result of the disparity in thermal conductivities between graphene and Al_2_O_3_. Given the significantly lower thermal conductivity of the Al_2_O_3_ substrate compared to graphene, the heat flow within the graphene skin experiences minimal variation during transmission. Consequently, the heat flux initially decreases and then increases from the heat source to the cold source, corresponding to the trend of the skin's cross‐sectional area, which enlarges initially and then diminishes. When the graphene thickness was set at 100 nm, the heat conduction pathways were more pronounced in Figure S23 (Supporting Information). In actual application scenarios, Gr‐skinned Al_2_O_3_ powders are filled in the polymer matrix to the maximum amount, in which the powders are in contact with each other. When the filling amount is very small, the real contact and interaction between the thermally conductive filler cannot be formed. The loading of Gr‐skinned Al_2_O_3_ powders in our TIM was 95.3 wt.%, which led to a real interaction between the thermal conductive filler and a thermal conduction chain or network being formed in the system, which will be further discussed in **Figure** **4**. To further analyze the heat transfer between connected powders, we constructed the heat flux of stacked Gr‐skinned Al_2_O_3_ powders with a 10.1 nm‐thick graphene layer (Figure S24, Supporting Information). The simulation results show that the heat flux within the graphene skin is more than an order of magnitude higher than that within the Al_2_O_3_ powder, indicating that the graphene layers in contact between the stacked particles form a continuous high‐speed thermal conduction pathway. Additionally, we selected Al_2_O_3_ powders skinned with a 40 nm‐thick graphene layer for numerical simulations to analyze the heat transfer behavior of stacked particles (Figure 3f). It is evident that the heat flux within the graphene skin surpasses that within the Al_2_O_3_ powders by more than an order of magnitude, indicating that the graphene skins in contact with the stacked powders form a continuous high‐speed thermal conduction pathway. This is consistent with the results obtained from the simulation of Gr‐skinned Al_2_O_3_ powders with a 10.1 nm‐thick graphene layer. This study employs thermal conduction simulations of multiple Gr‐skinned Al_2_O_3_ powders to demonstrate that preferential heat pathways, which are formed by the graphene layers.The combination of machine‐learning‐based models and experimental results can lead to an unprecedented level of understanding of thermal transport properties and structure‐thermal property correlations of Gr‐skinned Al_2_O_3_ powders for further application, which will be further discussed in Figure 4. To further substantiate the elevated thermal conductivity of Gr‐skinned Al_2_O_3_ powders in practical thermal management applications, in addition to theoretical calculations, the powders were incorporated into a polymer matrix using a mature industrial formula to obtain Gr‐skinned Al_2_O_3_ based TIM and Al_2_O_3_ based TIM (see more in experimental details). While previous simulations predict enhanced thermal flux with increasing graphene layers (Figure 3d), practical TIM implementation reveals critical mechanical‐thermal tradeoffs. Excessive graphene thickness improves thermal flux but degrades conformal contact and elevates the hardness of TIM, resulting in contact resistance escalation. As a result, we chose 30‐layer graphene (10.1 nm, growth time: 30 min) as the optimal thickness, which leverages graphene's thermal transport without compromising TIMs' essential viscoelastic functionality. The contact angle and Brunner‐Emmet‐Teller (BET) surface area of the pristine Al_2_O_3_ and Gr‐skinned Al_2_O_3_ powder were characterized for formula modification (Figures S25 and S26, Supporting Information). The prepared material is transferred to a fully automatic two‐roll thermal calender in order to adjust the thickness and size before undergoing a curing process. It is noteworthy that the process is straightforward and efficient, allowing for the scalable production of a substantial number of Gr‐skinned Al_2_O_3_ based TIMs. Figure 4a demonstrates a photograph of a single 20 × 40 cm piece of Gr‐skinned Al_2_O_3_ based TIM, exhibiting a uniform thickness of 1.0 mm and a smooth surface, indicating significant potential for continuous production and industrial application (Figure S27, Supporting Information). It can also be further processed to the requisite dimensions for the intended practical application. The cross‐sectional SEM image of Gr‐skinned Al_2_O_3_ based TIM in Figure 4b demonstrated the impeccable continuous and uniform structure constructed by various particle sizes of Gr‐skinned Al_2_O_3_ powders. Figure 4c compares the through‐plane thermal conductivity and thermal resistance between the Gr‐skinned Al_2_O_3_ and Al_2_O_3_ based TIM using the heat flow method. The Gr‐skinned Al_2_O_3_ based TIM demonstrated a higher thermal conductivity of ≈6.44 W·m^−1^·K^−1^, representing a 48% increase compared to the Al_2_O_3_ based TIM (≈4.35 W·m^−1^·K^−1^) based on the American Society for Testing and Materials (ASTM) D5470 test method. The superior heat conduction performance can be attributed to the higher thermal conductivity of the continuous graphene skin, which forms a network in the system, and the heat generated can be dissipated more efficiently. This results in a lower thermal resistance of ∼ 0.32°C·W^−1^, which is 53% lower than that of pristine Al_2_O_3_ (≈0.49°C·W^−1^). Compared with the previously reported TIMs that added multi‐layer graphene as thermal conductive fillers (5.1 W·m^−1^·K^−1^).^[^ ^23^ ^]^ our scheme also shows superiority in terms of filling amount and thermal conductivity. In addition, Gr‐skinned Al_2_O_3_ powder with different growth times and the pristine powder were pressed with 10 t pressure to obtain powder tablets for Hot Disk analysis (the principles are demonstrated in Figure S28, Supporting Information). Compared to the pristine Al_2_O_3_ tablet (1.418 W·m^−1^·K^−1^), the thermal conductivity of Gr‐skinned Al_2_O_3_ powder tablets increased by ≈2.96%‐14.6% with growth time and then decreased slightly, which highlights the correlation between the thickness of graphene and the resulting thermal conductivity (Figure 4c left). The slight decrease at 60 min was speculated to be the result of the high mass and heat transfer that at some point, the growth rate of the graphene domains cannot keep up with the cracking rate of the carbon source CH_4_, consequently generating slight amorphous carbon instead of forming hexatomic rings (Figure S8d, Supporting Information). Moreover, the obtained TIM samples were placed on a constant temperature heating stage of 100 °C to monitor the difference in radiation temperature until reaching equilibrium. The infrared camera was positioned directly above the different samples, which were processed to be round and placed on the heating stage simultaneously for recording. As illustrated in the infrared image, the color of the Gr‐skinned Al_2_O_3_ based TIM rapidly transitions from dark blue to bright red, which is closer to the surface temperature of the heater, in comparison to the Al_2_O_3_ based TIM, for which the color of the thermal image changes at a much slower rate (Figure 4d). This demonstrates a notable disparity in the heat transfer capability. Figure 4e illustrates the variation in radiation temperature of the different TIMs with respect to the contact time of the heating plate. It shows the equilibrium radiation temperature of the Gr‐skinned Al_2_O_3_ based TIM was observed to be ≈98.2°C, which was ≈6.7°C higher than that of the Al_2_O_3_ based TIM (≈91.5°C) at 35 s.

To validate the performance of the Gr‐skinned Al_2_O_3_ based TIM for thermal management in modern electronics, a 50 W LED was selected as the heat source with an air‐cooled straight‐fin radiator to constitute the experimental platform. It can be seen in Figure S29 (Supporting Information), an infrared camera was positioned directly above the targeted samples to record the temperature variation of the LED. The TIMs were sandwiched between the LED and the radiator under packaging pressure. The control group was set up with the LED directly mounted on the radiator to estimate and compare the cooling performance. As the working time increased, the average temperature of the LED lamp increased gradually, finally reaching a state of stability. Figure 4f illustrates the temperature curves for the heating and cooling processes. With no TIM, the average temperature of the LED surface reached 103.1 °C at 300 s, while the temperatures of the Al_2_O_3_ based TIM and Gr‐skinned Al_2_O_3_ based TIM were 92.7 and 85.4°C, respectively. It can be observed that the Gr‐skinned Al_2_O_3_ based TIM resulted in a temperature drop of 17.7°C of the LED surface, demonstrating a markedly lower equilibrium temperature and considerably higher cooling rates, indicative of superior thermal performance. Furthermore, the steady‐state temperature also decreased by 7.3°C, demonstrating the excellent heat transfer that occurs along the graphene skin, in comparison to the Al_2_O_3_ based TIM. Figure 4g and Figure S30 (Supporting Information) demonstrate the temperature cloud diagrams of the LED when using no TIM, Al_2_O_3_ based TIM and Gr‐skinned Al_2_O_3_ based TIM during the heating and cooling processes, respectively. The Gr‐skinned Al_2_O_3_ powder‐based TIM maintained a stable physical performance over 500 cycles of the heating and cooling cycle tests, alternating between 40°C (low) and 100°C (high) to simulate typical operating conditions (Figure S31, Supporting Information). The results reflected its superior thermal stability at different temperatures during long‐term application, showing <3% thermal conductivity decay during a 500‐cycle thermal stability test. TIMs serve diverse application scenarios, ranging from insulation‐critical applications to situations where electrical conductivity is permissible. The measured thermal conductivity of Gr‐skinned Al_2_O_3_ based TIM (≈6.44 W·m^−1^·K^−1^) exhibited clear advantages in comparison to the previously reported Al_2_O_3_ based TIMs utilizing carbon nanotubes (CNTs), epoxy, or other strategies aimed at enhancing thermal conductivity, as depicted in Figure 4h (refer to Table S5, Supporting Information for a more comprehensive overview).^[^ ^51^, ^52^, ^53^, ^54^, ^55^, ^56^, ^57^, ^58^, ^59^, ^60^ ^]^

## 3 Conclusion

The utilization of graphene as a heat dissipation solution remains undoubtedly one of the most promising approaches, exerting a significant impact on the sustainable development of the electronics industry. We report a facile and reliable strategy to introduce a “Phonon Expressway” comprising high‐quality continuous graphene skins inside the thermal interface material. Instead of the conventional static CVD process, we took the route of the FB‐CVD process owing to the combined advantages of both fluidized bed and CVD, thereby facilitating good heat and mass transfer as well as precise control over the quality and layer number of graphene skin. By combining machine learning and theoretical calculation, it was discovered that Gr in‐plane transport and Gr/Gr interlayer transport were preferential. ANSYS was employed to calculate the temperature, heat flux, and heat flow of the Gr‐skinned Al_2_O_3_ powder to further demonstrate the preferential thermal conductivity of graphene skin in the composite. Our study demonstrates that the high‐quality, continuous, and conformal coverage of graphene skin on conventional engineering Al_2_O_3_ powder materials ensures efficient heat transfer due to the exceptional thermal conductivity of graphene, combined with the strong phonon coupling between graphene and Al_2_O_3_. The interlayer thermal conductance of O‐Gr/Gr is two orders of magnitude higher than the interfacial thermal conductance of Al_2_O_3_‐O/O without graphene. The calculated heat flux within the graphene skin in contact between stacked powders surpasses that within the Al_2_O_3_ powders by more than an order of magnitude, establishing a contiguous high‐speed thermal conduction pathway in the composite system. In addition, the Gr‐skinned Al_2_O_3_ powder‐based TIM demonstrated superior thermal conduction performance, with a reduction of 17.7°C when utilized in an LED apparatus. The measured thermal conductivity of the TIM (6.44 W·m^−1^·K^−1^) also exhibits a notable superiority compared to other published Al_2_O_3_ powder based TIMs. Beyond the demonstrated thermal performance enhancements, the FB‐CVD process exhibits exceptional batch‐to‐batch reproducibility. The achieved CV values (<0.09) for graphene quality metrics meet the industrial‐grade repeatability, holding the promise of achieving a viable pathway toward stable large‐scale mass production of Gr‐skinned Al_2_O_3_ powder from laboratory‐scale preparation. It may be a potent strategy to provide foundations and pave the way for the subsequent industrialization of this transfer‐free material for thermal management applications.

## 4 Experimental Section

### Synthesis of Gr‐Skinned Al_2_O_3_ Powder Materials by FB‐CVD Process

The spherical Al_2_O_3_ powder materials utilized in the present research were purchased from Ya'an Bestry Performance Material Co., Ltd., China, with an average particle size of 5 µm, 40 µm, and 70 µm, respectively, and a purity exceeding 99.93%. Gr‐skinned Al_2_O_3_ powder was synthesized through the high‐temperature FB‐CVD method employing an atmospheric pressure fluidized bed system provided by Jiangsu Weipu NanoTechnology Co., Ltd, China. The initial step involves introducing a single batch of 150 g Al_2_O_3_ powder into a gas distributor within the fluidized bed reactor via an automatic feed unit. Subsequently, a carrier gas (Ar, 1000 sccm, atm) was introduced from the bottom of the distributor while maintaining the powder's fluidized state through the action of flowing air. After the temperature was increased from room temperature to 1100°C, H_2_ (500 sccm, atm) was introduced for a 10‐min pretreatment to eliminate surface pollutants on the powder and effectively enhance the growth of graphene skin. The reactor was then charged with CH_4_ (300 sccm), H_2_ (300 sccm), and Ar (900 sccm) to initiate the deposition of graphene skin until growth completion. The reaction was typically maintained for 10–120 min, after which CH_4_ flow was ceased and heating was turned off. The furnace undergoes cooling under Ar protection, while the H_2_ flow stops at 800°C. Once the fluidized bed reactor reached room temperature, Ar flow was discontinued, and the resulting Gr‐skinned Al_2_O_3_ powder material was sealed for storage.

### Preparation of Gr‐Skinned Al_2_O_3_ Powder Based TIMs

The vinyl silicone oil (100 g) with a viscosity of 500 ± 30 mPa·s (25°C) was weighed using a precision electronic balance, and preheated at 120°C in the vacuum drying oven for 20 min to remove the tiny air bubbles in the vinyl silicone oil (90 g double‐capped vinyl silicone oil with vinyl content of 0.21 ± 0.02 wt.% and 10 g single‐capped vinyl silicone oil with vinyl content of 0.43 ± 0.02 wt.%). Then, Gr‐skinned Al_2_O_3_ powder with different particle sizes and 11.5 g Dynasylan 9116 were sequentially introduced into the silicone oil matrix, followed by thorough stirring to ensure uniform distribution (70 µm: 40 µm: 5 µm = 1200 g: 700 g: 400 g). Afterward, 0.15 g of 1‐ethynyl‐1‐cyclohexanol, 0.37 g of platinum catalyst, and 2.0 g polymethylhydrosiloxane were subsequently added and stirred manually before using a double planetary power mixer to make the mixture homogeneous. Following thorough mixing, the filler was subjected to vacuum treatment in a vacuum drying oven. The prepared material was subsequently transferred to a fully automated two‐roll thermal calender equipped with release film on both sides, where the thickness was adjusted to 1.0 mm for the calendering process. The rolled thermal silica gel was cured at a temperature of 120°C for a duration of 10–15 min in a tunnel oven to obtain Gr‐skinned Al_2_O_3_ powder‐based TIM. Additionally, the control sample was prepared by utilizing the original untreated Al_2_O_3_ powder through the same process.

### Transfer Process of Graphene Skin

In order to remove the Al_2_O_3_ substrate, HCl was utilized as the etching agent for wet transfer. The 0.2 g Gr‐skinned Al_2_O_3_ powder was dispersed in a 20 mL solution of 12 mol·L^−1^ HCl for 72 h to eliminate the internal Al_2_O_3_ powder. Once complete etching of the Al_2_O_3_ core was achieved, the remaining graphene skin was subjected to three rounds of cleaning with deionized water before being transferred onto the TEM grid. Subsequently, HR‐TEM characterization can be performed on the obtained samples after atmospheric drying.

### Characterization

SEM images were obtained using an FEI Quattro S scanning electron microscope (Acceleration voltage 10 kV). The thickness of the graphene skin was measured by FIB (Helios UX) and TEM (Thermo Fisher Talos F200X G2). STEM images were acquired using the aberration‐corrected TEM (JEOL ARM300F2, at 300 keV electron energy). The bonding forms on the surface were analyzed using XPS (Thermo Fisher Nexsa G2). Raman characterization was conducted to analyse the chemical composition and graphene quality (Horiba, LabRAM HR‐800, laser excitation wavelength of 532 nm, 50 × objective lens). The carbon content was assessed using Thermogravimetric Analysis (Mettler Toledo TGA2). XRD characterization was conducted using Bruker D8 ADVANCE. BET surface area was analyzed using Micromeritics ASAP2460. Hot Disk TPS3500 was used to collect the thermal conductivity of Gr‐skinned Al_2_O_3_ and pristine Al_2_O_3_ powder tablets. Xiangtan Xiangyi Instrument Co., Ltd. DRL‐V Thermal Conductivity Tester (Heat flux) was used to detect the thermal conductivity and thermal resistance of TIMs. The infrared images were collected by a Fluke Ti 10 infrared camera. The graphene coverage was estimated via Photoshop to calculate the cladding rate.

## Conflict of Interest

The authors declare no conflict of interest.

## Supporting information



Supporting Information

Supplemental Video 1

Supplemental Video 2

Supplemental Video 3

Supplemental Video 4

## Data Availability

Research data are not shared.
