# Supplementary material for: Controlled Growth of Graphene‐Skinned Al2O3 Powders by Fluidized Bed‐Chemical Vapor Deposition for Heat Dissipation
Source: Adv Sci (Weinh). 2025 Jul 29;12(40):e03388. doi: 10.1002/advs.202503388 (PMC12561332; doi:10.1002/advs.202503388)
Supplement: Supplementary file 1 — Supporting Information [file ADVS-12-e03388-s001.docx]

Supporting Information

Controlled Growth of Graphene-skinned Al_2_O_3_ Powders by Fluidized Bed-Chemical Vapor Deposition for Heat Dissipation

*Yuzhu Wu^a,b^, Zhifeng Sun^b,c^, Ningning Liu^d^, Zhong Wang^b,e^, Yueming Hu^b,f^, Tianqi Bai^b,f,g^, Tao Wang^g^, Jingyang Chen^b^, Xiaopan Qiu^b^, Xudong Zhang^h,i^, Fushun Liang^b,f^, Dongcheng Jiao^b^, Dan Li^b^, Lishuo Han^b^, Wenhu Wang^b,j^, Qin Xie^a,b^, Ronghua Zhang^b^, Ali Cai^b^, Yuqi Xia^b^, Haonan Zhai^b^, Zhong-zhen Yu^c^, Yue Qi^b^, Chu Wang^k^, Peng Gao^b,f,g,l,m^, Xiucai Sun^b*^, Bingyang Cao^d*^, Yuqing Song^b*^, Zhongfan Liu^a,b,f*^*

**Figure S1. Cross-sectional transmission electron microscope (TEM) and fast Fourier transform (FFT) images of α-Al_2_O_3_ powder substrate.**

**
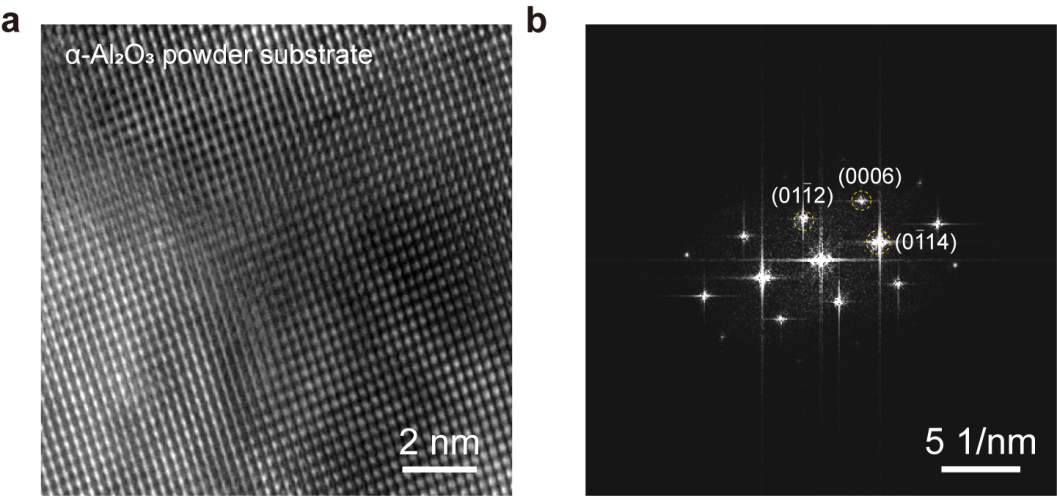
**

**Figure S1.** Cross-sectional TEM and FFT images of α-Al_2_O_3_ powder substrate after focused ion beam (FIB) treatment.

**Figure S2. Illustration of fluidized bed-chemical vapor deposition (FB-CVD) equipment and the typical growth procedure of graphene-skinned (Gr-skinned) Al_2_O_3_ powder materials.**

The preparation of Gr-skinned powder composites in previous research primarily relied on traditional chemical vapor deposition (CVD) methods. However, considering the unique structure and properties of powder materials, industrial-scale production inevitably faces challenges such as low yield, subpar quality, high production cost, and significant energy consumption. As a result, we proposed the FB-CVD method, which has the advantages of a high heat transport rate, a homogenous concentration field, and important applications in the preparation of novel graphene composite materials. The diagram of the equipment's structure is depicted in **Figure S2a.** The equipment primarily consists of a fluidized bed reactor, a heating furnace, an intelligent control panel, and an automatic feeding and discharging system. The utilization of a micro-porous plate gas distributor is noteworthy as it enhances the stability of the gas-solid flow, thereby increasing the conversion frequency between the thin phase and thick phase in the central and bottom regions of the fluidized bed reactor. This results in a more uniform distribution of the gas phase.

Simultaneously, to address the issue of controllable and uniform growth of graphene skin on spherical powder substrates while considering the cost and yield of mass material preparation, we devised a fully automatic feeding and discharge system that effectively reduces energy consumption and labor costs through its control device. We incorporated a high-temperature resistant coating inside the storage tank so that when the temperature reaches the set parameter (450℃) during cooling, the target material is collected into the storage tank, and a new batch of original Al_2_O_3_ powder material is blown into the reactor to shorten cooling time. The complete process for graphene skin growth is illustrated in **Figure S2b**.

**
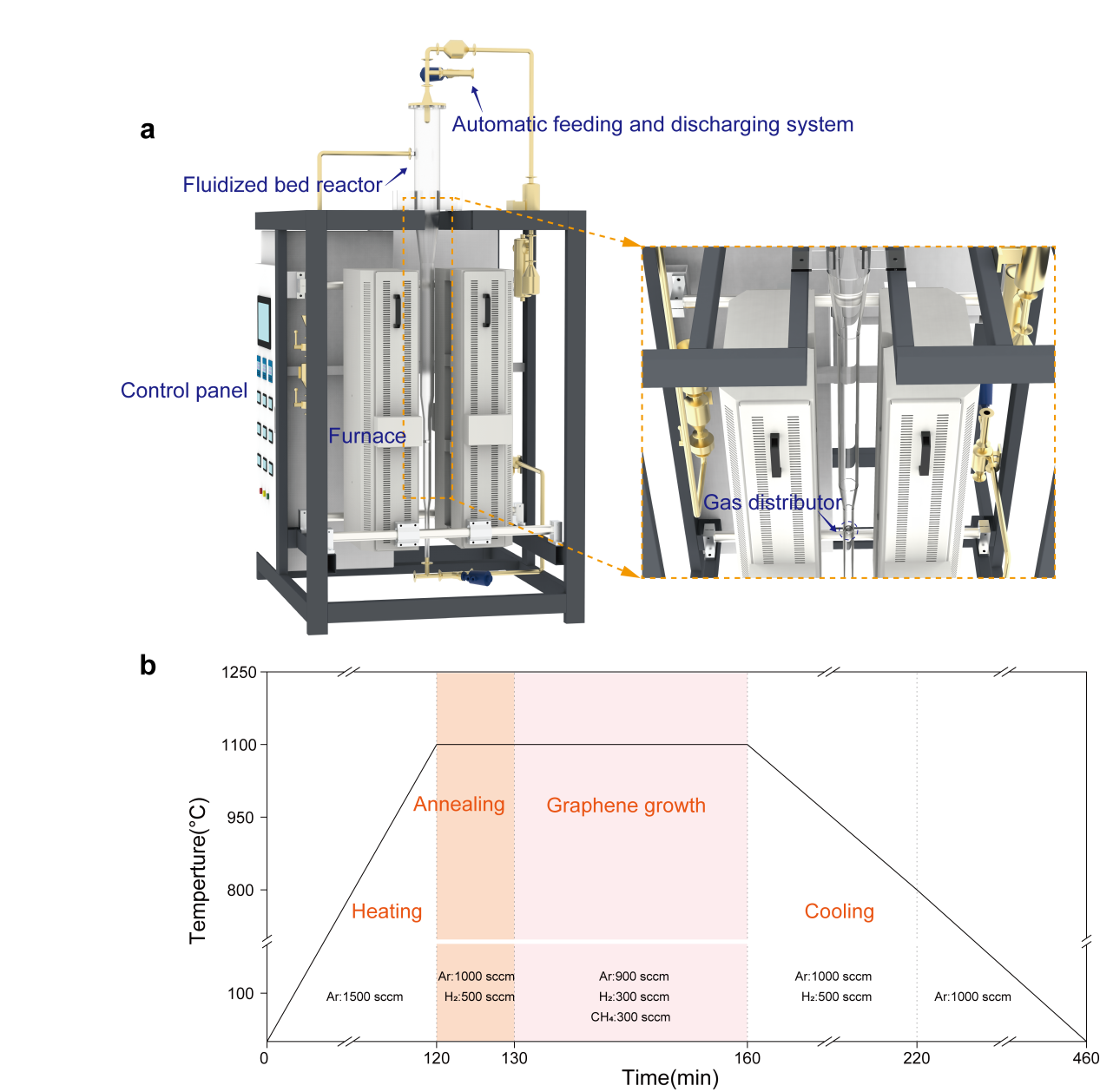
**

**Figure S2.** Illustration of fluidized bed-chemical vapor deposition equipment and the typical growth procedure of Gr-skinned Al_2_O_3_ powder materials. a) Schematic diagram of FB-CVD equipment for the synthesis of graphene skin. Left: The laboratory-scale fluidized bed with a single batch capacity of 150-200 g, including the fluidized bed reactor, heating furnace, intelligent control panel, automatic feeding and discharging system, etc. Right: Magnified view of fluidized bed reactor. The micro-porous plate gas distributor is used to support the powder materials and uniformly distribute the gas flow to improve the fluidization state. b) Typical growth procedure of Gr-skinned Al_2_O_3_ powder materials. The growth process of graphene skin was carried out under atmospheric pressure. The furnace was heated to 1100℃ and maintained there for 10 min for the pretreatment process (Ar 1000 sccm and H_2_ 500 sccm). Afterwards, CH_4_ (300 sccm) was introduced into the reactor for graphene skin deposition.

**Figure S3. The temporal variation of the volume fraction distribution of Al_2_O_3_ powder in a fluidized bed.**

The particles can be classified into four categories according to Geldart classification (1973): A, B, C, and D. Among these categories, Class B particles exhibit a closer resemblance to Al_2_O_3_ powder material due to their average particle size range of 40 to 500 μm and density range of 1400 to 4000 kg·m^-3^. ^[1]^

In the study of hydrodynamic behavior within the gas and solid fluidized beds using CFD, three-dimensional (3D) models are often closer to the actual situation than two-dimensional (2D) models.^[2]^ However, 3D models require more computational resources and time, and many researchers prefer to simplify the actual bed into a 2D model.^[3]^ 2D simulations cannot completely replace 3D simulations, but they can still be used in qualitative studies to provide valuable information for understanding the flow state. Therefore, based on the actual model size, a 2D fluidized bed model was established, and a quadrilateral structured grid was employed to mesh the fluidized bed. Due to the absence of internal components in the fluidized bed, a simplified 2D axisymmetric structure could be utilized for modelling purposes, resulting in a significant reduction in computational workload and time consumption. As depicted in **Figure S3a**, SpaceClaim software was employed to construct the solid geometry model of the 2D fluidized bed, while Workbench grid division facilitated grid generation. The maximum skewness value obtained from **Table S1** is 0.657. Subsequently, the generated grid file was imported into Fluent 2022R2 where it underwent thorough checking to ensure that no negative volume grids were produced and that overall grid quality met stipulated requirements.

In Eulerian multiphase flow, the gas and solid powder motions are solved within the Euler reference frame. The mass and momentum equations are separately solved for each phase. However, this approach does not allow for tracking individual powders to accurately represent bubble formation beyond a specific volume fraction. The primary objective of this model is to solve the homogeneous mean Navier-Stokes equation. Gidaspow^[4]^ and Ding^[5]^ successfully simulated gas-solid fluidization and effectively predicted the hydrodynamic behavior of bubbling beds. The simulation content of this study includes mathematical equations as follows:

- 1. Fundamental equations of Euler-Euler two-fluid model

The Euler-Euler multiphase flow model treats each phase as an interpenetrating continuum. This model solves the mass, momentum and energy transport equations of each phase when all phases share a single pressure field. The Eulerian mean value of the transport equation leads to additional interphase interactions. These interactions require closed models such as drag, lift, interphase heat transfer, etc.

The continuity equation is expressed as:

$\begin{matrix} \frac{\partial(\rho_{g}\varepsilon_{g})}{\partial t}+\nabla(\rho_{g}\varepsilon_{g}\overset{\to}{v_{g}})=0 \\ \frac{\partial(\rho_{s}\varepsilon_{s})}{\partial t}+\nabla(\rho_{s}\varepsilon_{s}\overset{\to}{v_{s}})=0 \end{matrix}$ (1.1)

ε —— Phase volume fraction;

ρ —— Phase density (kg·m^-3^);

v —— Speed (m·s^-1^);

g, s —— Denote the gas phase and the particle phase, respectively.

Gas and powder are mixed with each other, and the volume fraction of the two phases in each cell should be added to 1.

$\varepsilon_{\text{g}}+\varepsilon_{s}=1$ (1.2)

The momentum equation (Navier-Stokes equation) is expressed as:

$\frac{\partial(\rho_{g}\varepsilon_{g}\overset{\to}{v_{g}})}{\partial t}+\nabla(\rho_{g}\varepsilon_{g}\overset{\to}{v_{s}}\overset{\to}{v_{g}})=\beta(\overset{\to}{v_{s}}-\overset{\to}{v_{g}})-\varepsilon_{g}\nabla P+\varepsilon_{g}\rho_{g}g+\nabla\varepsilon_{g}\tau_{g}$ (1.3)

$\frac{\partial\rho_{s}\varepsilon_{s}{\overset{\to}{v}}_{s}}{\partial t}+\nabla(\rho_{s}\varepsilon_{s}\overset{\to}{v_{s}}\overset{\to}{v_{g}})=\beta(\overset{\to}{v_{g}}-\overset{\to}{v_{s}})-\varepsilon_{s}\nabla P+\varepsilon_{s} (\rho_{s-}\rho_{g})+[\nabla\varepsilon_{s}\tau_{s}-G_{s}\nabla\varepsilon_{s}]$ (1.4)

$\tau_{k}=\mu_{k} (\nabla\overset{\to}{v_{k}}+{(\nabla\overset{\to}{v_{k}})}^{T})(\lambda_{k}-\frac{2}{3}\mu_{k})\nabla\cdot\overset{\to}{v_{k}}I$ (1.5)

g —— Acceleration of gravity (m·s^-2^);

G_s_ —— Particle corresponding force coefficient;

I —— Unit stress tensor;

P —— Pressure (Pa);

β —— Gas-solid momentum transfer coefficient;

k —— Kinetic viscosity (Pa·s);

τ —— Pressure strain tensor (N·m^-2^).

- 1. Gidaspow drag model

The Gidaspow model is suitable for discrete solids with high particle loads, i.e. fluidized beds. The most commonly used formulas are the Ergun equation (for regions with high concentrations of solid particles) and the modified Stokes Law (for regions with low concentrations). The conversion volume fraction ε is used as the basis for switching between these formulas,

$\beta=\{\begin{matrix} \frac{3}{4}C_{D}\frac{\varepsilon_{s}\varepsilon_{g}\rho_{g}}{d_{s}}|\overset{\to}{v_{s}}-\overset{\to}{v_{g}}|\varepsilon_{g}^{-2.65} & \varepsilon_{g}>0.8 \\ 150\frac{\varepsilon_{s} (1-\varepsilon_{g})}{\varepsilon_{g}d_{s}^{2}}+1.75\frac{\rho_{g}\varepsilon_{s} |\overset{\to}{v_{s}}-\overset{\to}{v_{g}}|}{d_{s}} & \varepsilon_{g}\leq0.8 \end{matrix}$ (1.6)

C_D_ —— Drag coefficient;

μ_g_ —— Vapor phase viscosity (Pa·s).

1.3 Simulation parameter setting

The specific setting parameters of this study are as follows: the gas flow rate is 1.5 L·min^-1^ into the fluidized bed and the powder receiving region, and the internal fluidization state above the position of the sieve plate is calculated. At the same time, the order of Al_2_O_3_ powder in the fluidized bed is moderate, and the powders are set to the spherical state with a density of 4.0 g·cm^-3^. The specific heat capacity is 750 J·kg^-1^ and the thermal conductivity is 12.9 W·m^-1^·K^-1^. The particle size is set as 40 μm and 70 μm, respectively. The gas was a mixture of CH_4_, H_2_ and Ar at the temperature of 1100℃. The density of the mixture was 0.2224 g·L^-1^ according to the ratio (2.1).

$\rho_{\text{mixture}}=\rho_{A\text{r}}\cdot\varphi_{A\text{r}}+\rho_{CH_{4}}\cdot\varphi_{\text{CH}_{4}}+\rho_{H_{2}}\cdot\varphi_{H_{2}}$ (2.1)

$\rho$ —— Gas density;

$\varphi$ —— Gas volume fraction.

and the velocity inlet is set at the position of the screen plate at 0.234 m·s^-1^ according to the gas volume of 1.5 L·min^-1^. The wall temperature is set to a fixed temperature of 1100℃.

The solid and gas phase parameters are set according to the actual situation, and the initial solid volume fraction is set at the position of the powder area, which is 0.43. The rest of the reaction zone is initially filled with gas. In the simulation process, the gas phase is specified as a no-slip boundary, while the solid particle phase is set as a local slip boundary with a specular reflection coefficient of 0.6. In order to avoid the influence of the backflow, the pressure outlet boundary is not set. The total simulation time is 4 s and the calculated time step is 0.001 s.

The simulation results indicate that the fluidized state corresponds to a bubbling fluidized bed with powders measuring 40 μm in size (**Figure S3b**). Initially, gas enters the fluidized bed and generates bubbles at its base. As the ascent progresses, these bubbles gradually enlarge while the powders move upwards due to gas resistance. During the upward extension of the jet, particle aggregation occurs within a central agglomeration area before gravity causes them to descend, resulting in bubble bursting. Subsequently, as the powders fall again under the gas influence, the bed expands once more until it transitions into a stable state.

The fluidized state can be defined as a bubbling fluidized bed at a particle size of 70 μm, as depicted in the **Figure S3c**. In a bubbling fluidized bed, the bubble size exhibits a linear increase with both bed height and gas velocity, demonstrating minimal dependence on particle size. Consequently, the primary distinction among beds of different particle sizes lies in their respective heights. For instance, a fluidized bed comprising particles sized at 40 μm possesses an approximate height of 356 mm, while that with particles sized at 70 μm has an approximate height of 244 mm.


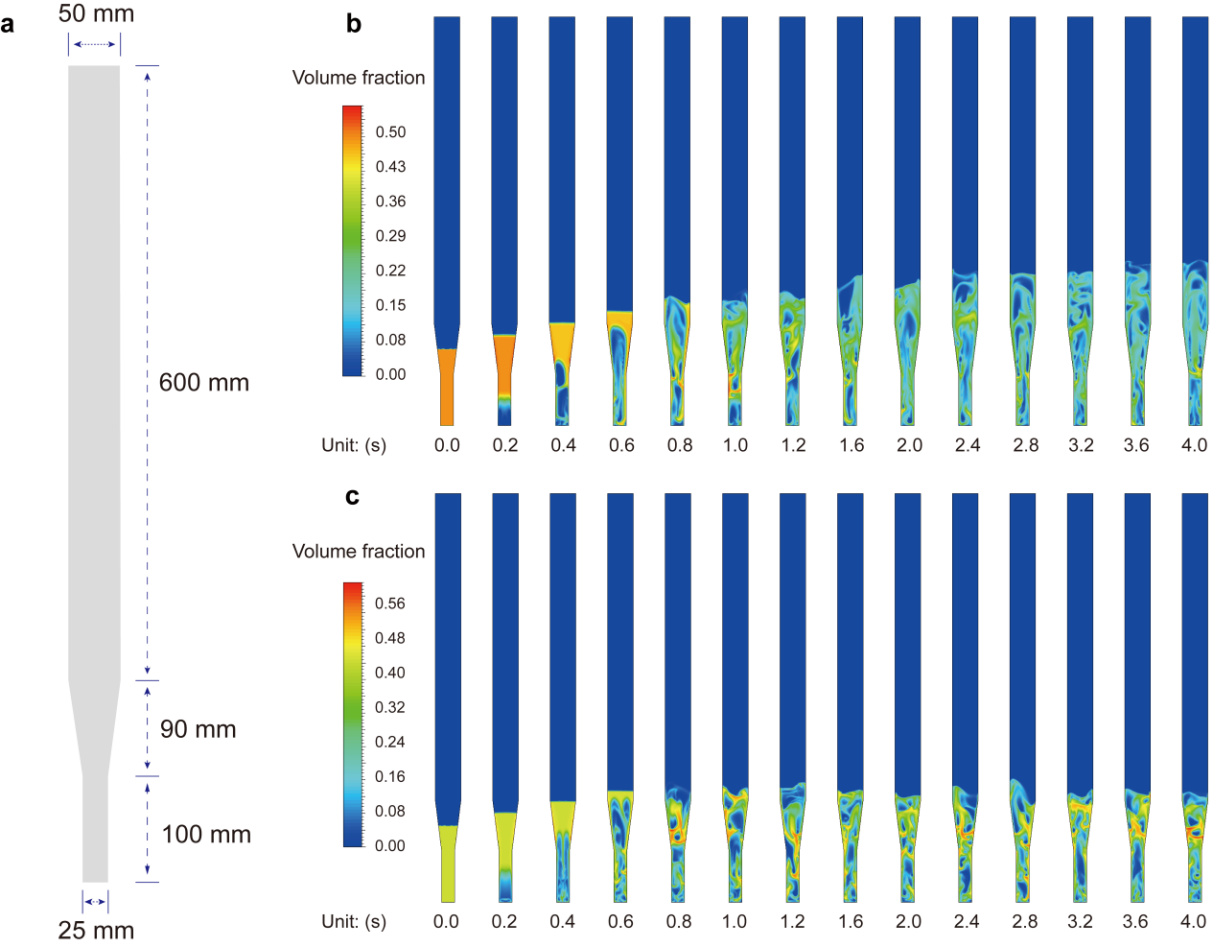


**Figure S3.** The temporal variation of the volume fraction distribution of Al_2_O_3_ powder in a fluidized bed. a) The demonstration of the fluidized bed model. b-c) The diagram of the temporal variation of the volume fraction distribution of Al_2_O_3_ powder in a fluidized bed of 40 μm b) and 70 μm c) particle size, respectively.

**Figure S4. The temporal variation of the pressure** **distribution of Al_2_O_3_ powder in a fluidized bed.**

To investigate the temporal variation of pressure distribution in the fluidized bed containing Al₂O₃ powder, four monitoring points were strategically positioned at distances of 0.0 m, 0.1 m, 0.2 m, and 0.3 m along the model (**Figure S4a**).

In the process of fluidization, the bubbles undergo a transition from a regular shape to an irregular shape and consistently exhibit tendencies towards splitting and coalescence. Consequently, there are fluctuations in pressure at different time intervals. As depicted in **Figure S4**, the pressure within the fluidized bed is correlated with its state of fluidization. Notably, when comparing particle sizes of 40 μm and 70 μm, it can be observed that the pressure fluctuation is more stable for particles with a size of 40 μm.


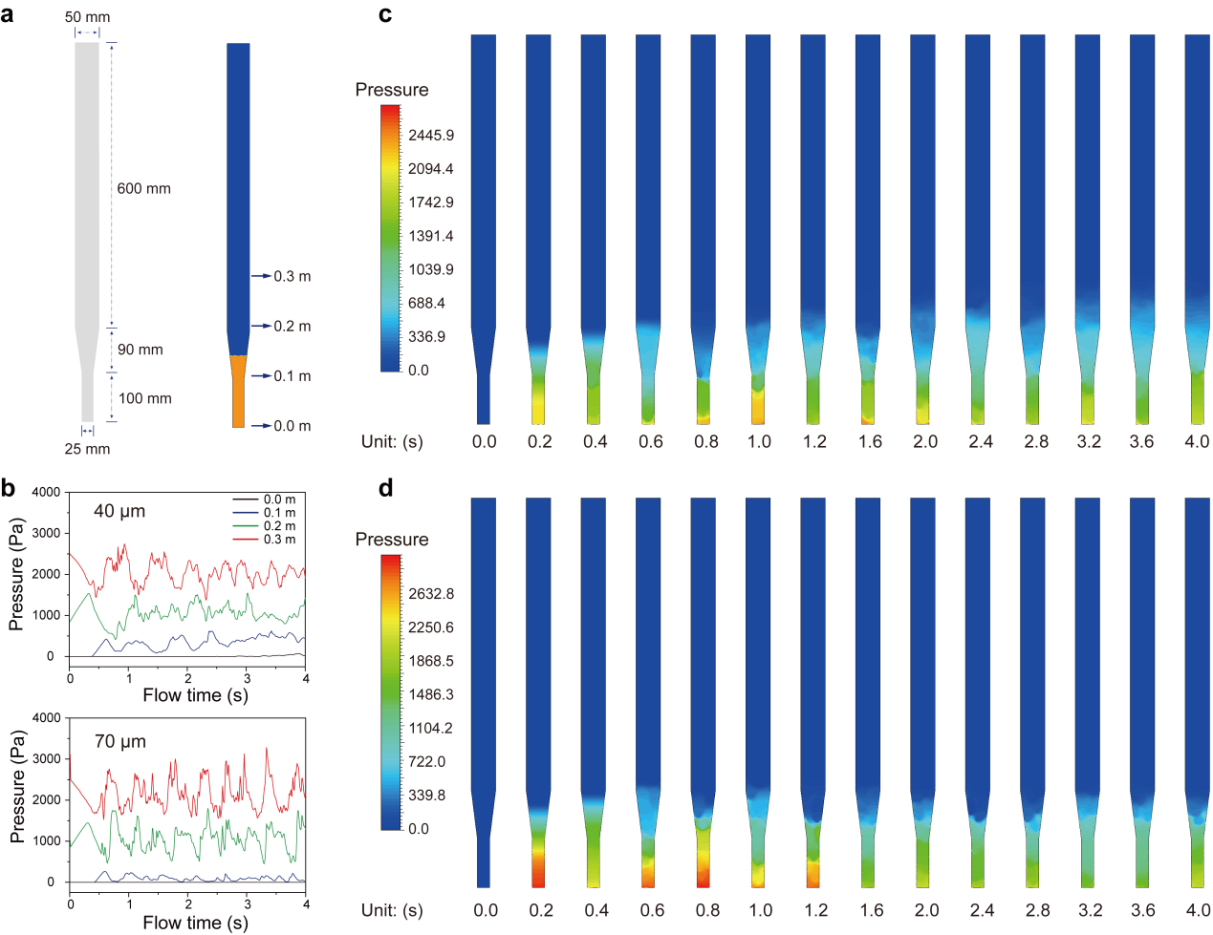


**Figure S4.** The temporal variation of the pressure distribution of Al_2_O_3_ powder in a fluidized bed. a) Illustration of four monitoring points, positioned in accordance with the specified model, at the following coordinates: 0.0 m, 0.1 m, 0.2 m and 0.3 m, respectively. b**)** The temporal variation of the pressure distribution of four monitoring points in a fluidized bed of 40 μm and 70 μm particle size. c-d) Diagram of the corresponding temporal variation of the pressure distribution of Al_2_O_3_ powder in a fluidized bed of 40 μm (c) and 70 μm (d) particle size, respectively.

**Figure S5. Photograph of Al_2_O_3_ powder and Gr-skinned Al_2_O_3_ powder.**


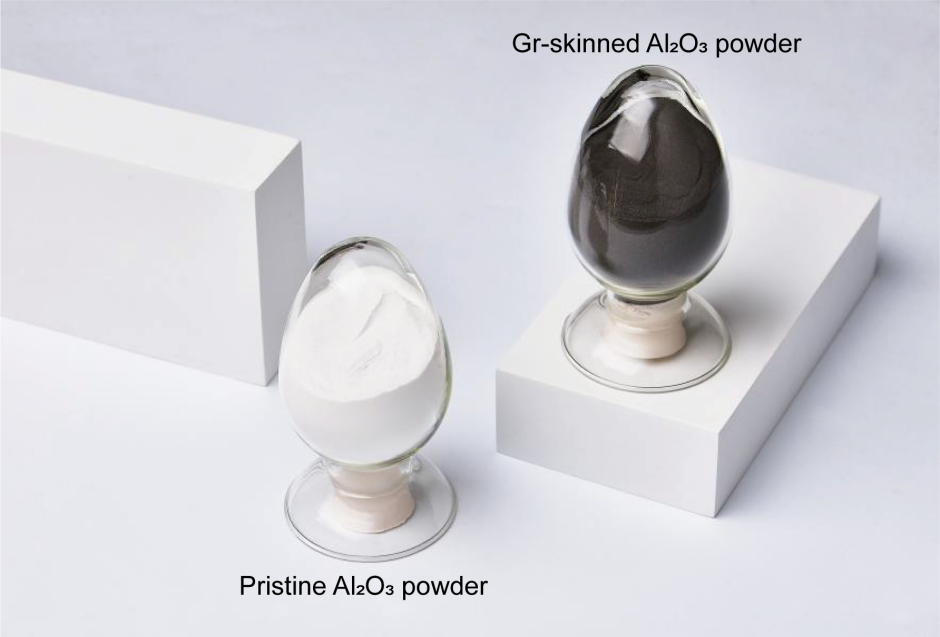


**Figure S5.** Photograph of Al_2_O_3_ powder (left) and Gr-skinned Al_2_O_3_ powder (right).

**Figure S6. Typical Scanning Electron Microscopy (SEM) images of pristine Al_2_O_3_ powder materials and Gr-skinned Al_2_O_3_ powder materials.**

The SEM images in **Figure S6** depict the characteristic morphology of a single Al_2_O_3_ powder before and after graphene growth. The growth of graphene skin results in the preservation of a spherical morphology and surface structure for Al_2_O_3_ powder, with each powder exhibiting a uniform and conformal layer of graphene covering.


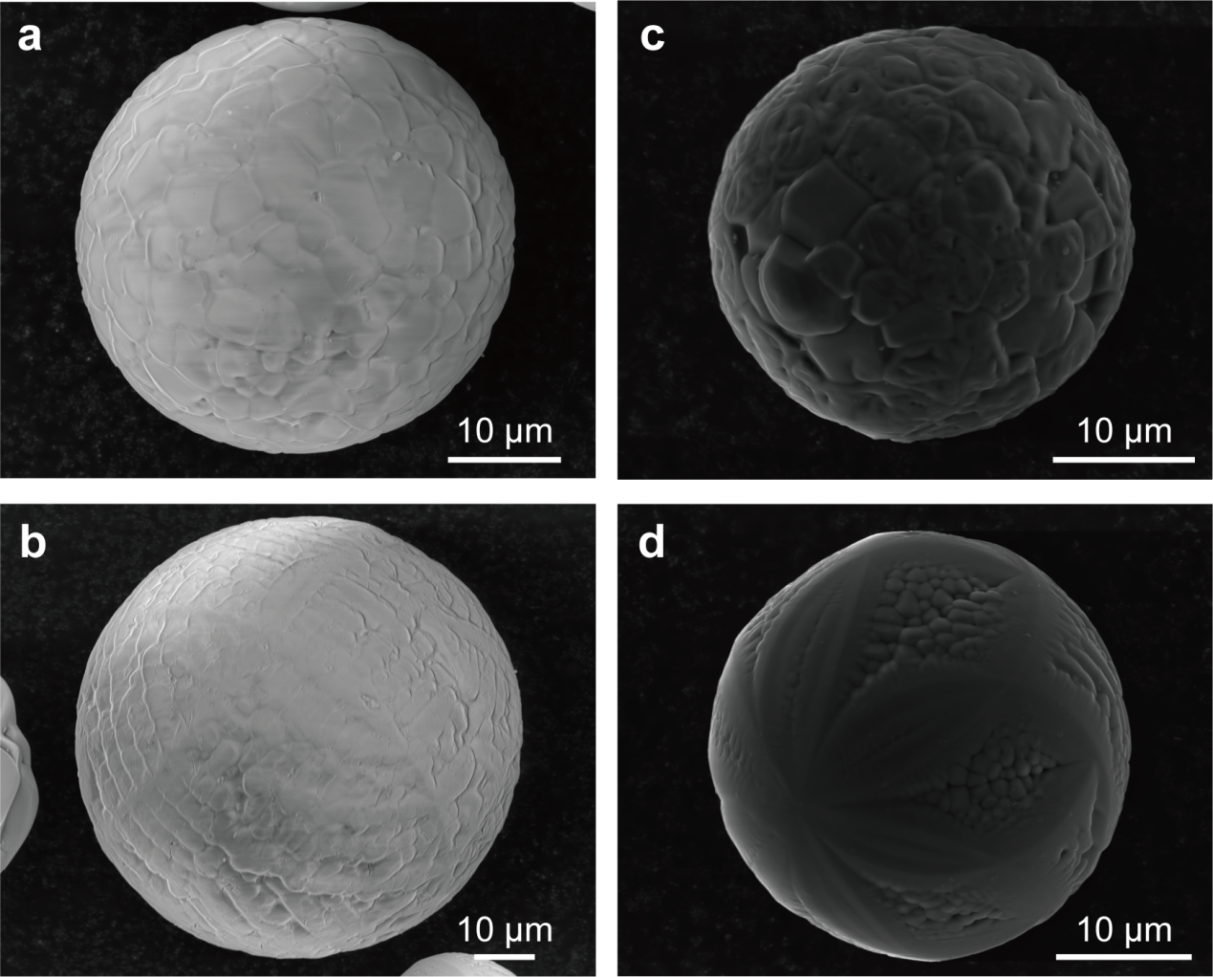


**Figure S6.** Typical SEM images of pristine Al_2_O_3_ powder materials and Gr-skinned Al_2_O_3_ powder materials. a-b) SEM of pristine Al_2_O_3_ powder. c-d) SEM of Gr-skinned Al_2_O_3_ powder (1100°C, atm, Ar/H_2_/CH_4_: 900/300/300 sccm, 30 min).

**Figure S7. SEM images of 40 μm Gr-skinned Al_2_O_3_ powder materials.**

**
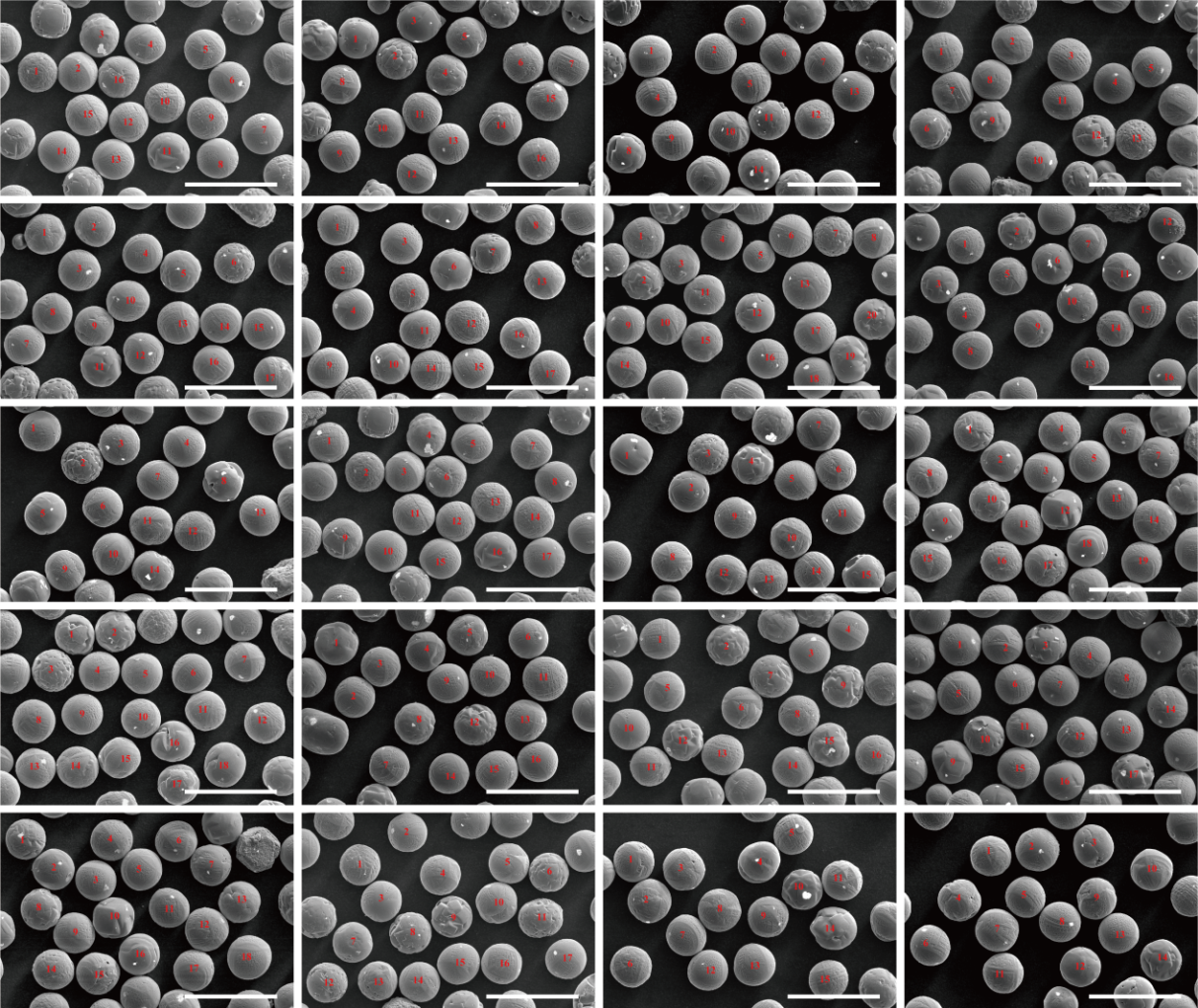
**

**Figure S7.** SEM images of 40 μm Gr-skinned Al_2_O_3_ powder materials. The sampling procedure was conducted in accordance with the method specified in the national standard GB/T 41978-2022 to ensure the randomness and reliability of the relevant tests. In terms of Gr-skinned Al_2_O_3_ powder materials, a clear distinction can be observed in the surface morphology between the region coated with graphene and the region devoid of such a coating. When secondary electrons interact with the powder sample within SEM, varying numbers of secondary electrons are detected by the SEM detector, resulting in contrasting patterns observed in the image. Each SEM image contains approximately 13-20 powders, which are identified based on their respective serial numbers. The white spots depicted in the SEM image represent areas that lack graphene coating. Scale bar, 100 μm.

**Figure S8. High-resolution TEM (HR-TEM) images of different graphene layers on Al_2_O_3_ powder.**


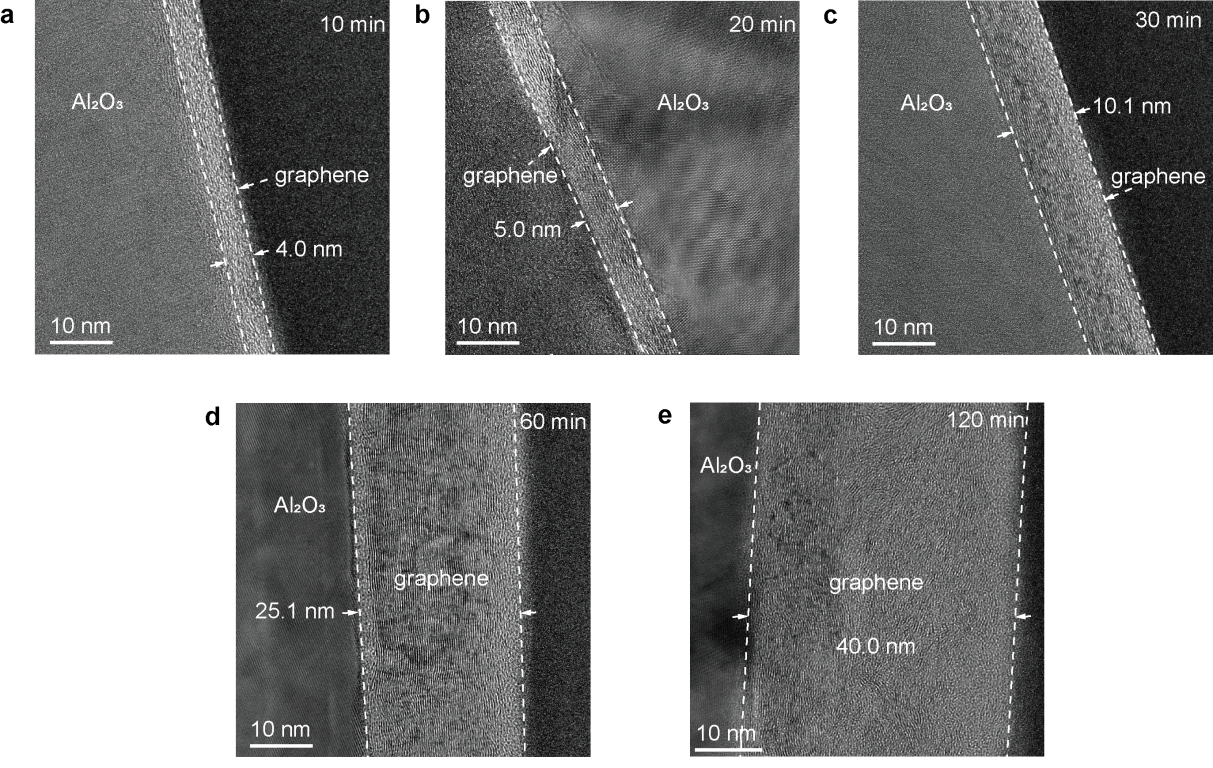


**Figure S8.** HR-TEM images of different graphene layers on Al_2_O_3_ powder of different growth duration (10, 20, 30, 60, 120 min). Detailed growth conditions: 1100°C, 900 sccm Ar, 300 sccm H_2_, and 300 sccm CH_4_.

**Figure S9. Representative HAADF-STEM (High-Angle Annular Dark-Field Scanning Transmission Electron Microscopy) image and energy-dispersive X-ray spectroscopy (EDS) maps of Gr-skinned Al_2_O_3_ powder materials after FIB treatment.**


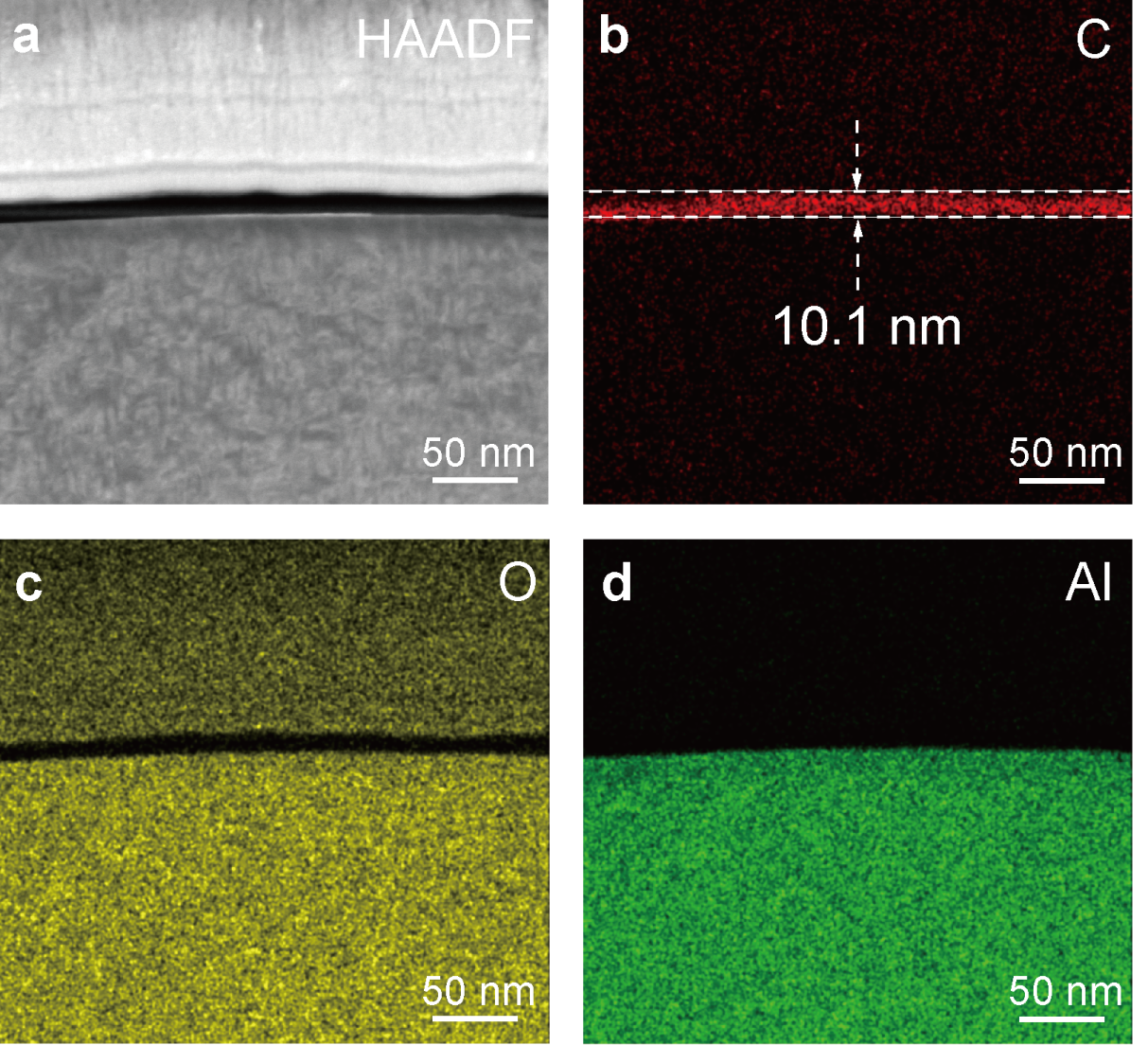


**Figure S9.** a) Typical HAADF-STEM image of Gr-skinned Al_2_O_3_ powder materials after FIB treatment. Elemental mapping of b) Al c) O, and d) C of cross-sectional Gr-skinned Al_2_O_3_ powder. The graphene skin layer was approximately 10.1 nm thick. Detailed growth conditions: 1100°C, 30 min, 900 sccm Ar, 300 sccm H_2_, and 300 sccm CH_4_.

**Figure S10. High resolution transmission electron microscope (HRTEM) images and EDS mapping of the etched Gr-skinned Al_2_O_3_ powder.**

The etched Gr-skinned Al_2_O_3_ powder was transferred onto a TEM grid for further analysis. As illustrated in **Figure S10**, powder exhibiting incomplete etching with a residual Al₂O₃ core (appearing dark in a BF-TEM image) and in conformal contact with continuous graphene skin was observed. This finding was further substantiated by EDS maps, which revealed the presence of Al and O elements within the core.


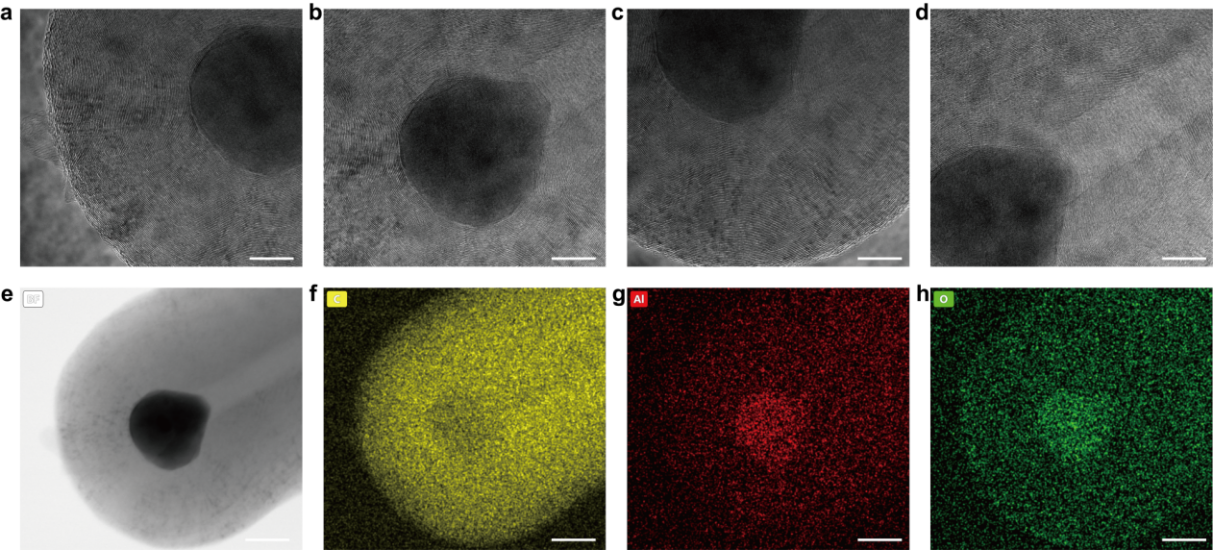


**Figure S10.** a-d) HRTEM images of the etched 5 μm Gr-skinned Al_2_O_3_ powder (See more experimental details in Methods). e) BF-TEM image and elemental mapping of f) C g) Al, and h) O of the etched Gr-skinned Al_2_O_3_ powder. Scale bar, 10 nm.

**Figure S11. The thermogravimetric analysis (TGA) graph and the differential thermogravimetric analysis (DTG) graph of the Gr-skinned Al₂O₃ powder with different numbers of graphene layers.**

TGA and DTG can be employed as a preliminary tool for the qualitative and quantitative analysis of graphene. The number of graphene layers was determined using a focused ion beam transmission electron microscope (FIB-TEM). Samples comprising different numbers of graphene layers were subjected to thermal decomposition in air from 25°C to 1000°C to investigate the layer-dependent thermal characteristics. The thermal patterns illustrated in the graphs demonstrate that the graphene skin on the Al_2_O_3_ powder was completely oxidized in the air atmosphere at 1000°C, leaving the Al_2_O_3_ powder behind. The proportion of graphene by mass is approximately 0.15-1.53% (12-75 layers). The DTG peaks can be attributed to the combustion of carbon in an air atmosphere. As illustrated in the DTG graphs, the temperature of maximum mass change rate (T_max_) is a distinct marker of carbon combustion in air. T_max_ can be attributed to the maximum external heat energy required to overcome the bonding within the carbon lattice structure. T_max_ increased significantly (712-804℃) with an increase in the number of graphene layers.


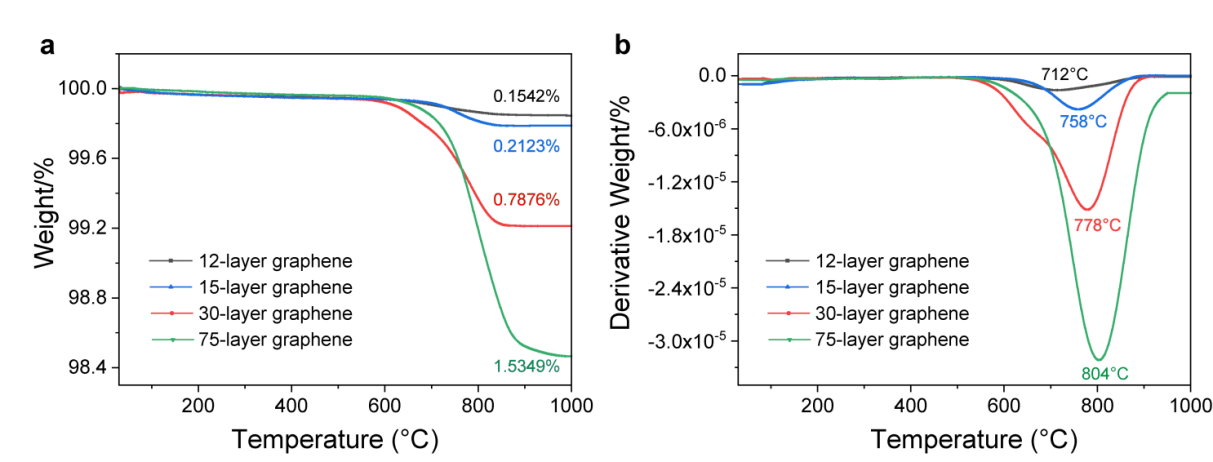


**Figure S11.** a) TGA graph and the b) DTG graph with the respective DTG peak values of the Gr-skinned Al₂O₃ powder with different numbers of graphene layers.

**Figure S12.** **Representative Raman spectra of p****ristine Al_2_O_3_ powder and** **Gr-skinned Al_2_O_3_ powder.**

The quality of the as-fabricated Gr-skinned Al_2_O_3_ powder was investigated using Raman spectroscopy. As depicted in **Figure S12**, the D-peak at approximately 1350 cm^-1^ is commonly attributed to the disorder-induced vibration peak of graphene, resulting from lattice motion away from the centre of the Brillouin zone. The I_D_/I_G_ ratio (the strength ratio between the D-peak and G-peak) serves as a significant indicator for assessing the defect density within the graphene layer and can be employed as a reliable criterion for analyzing the quality of Gr-skinned Al_2_O_3_ powder materials.


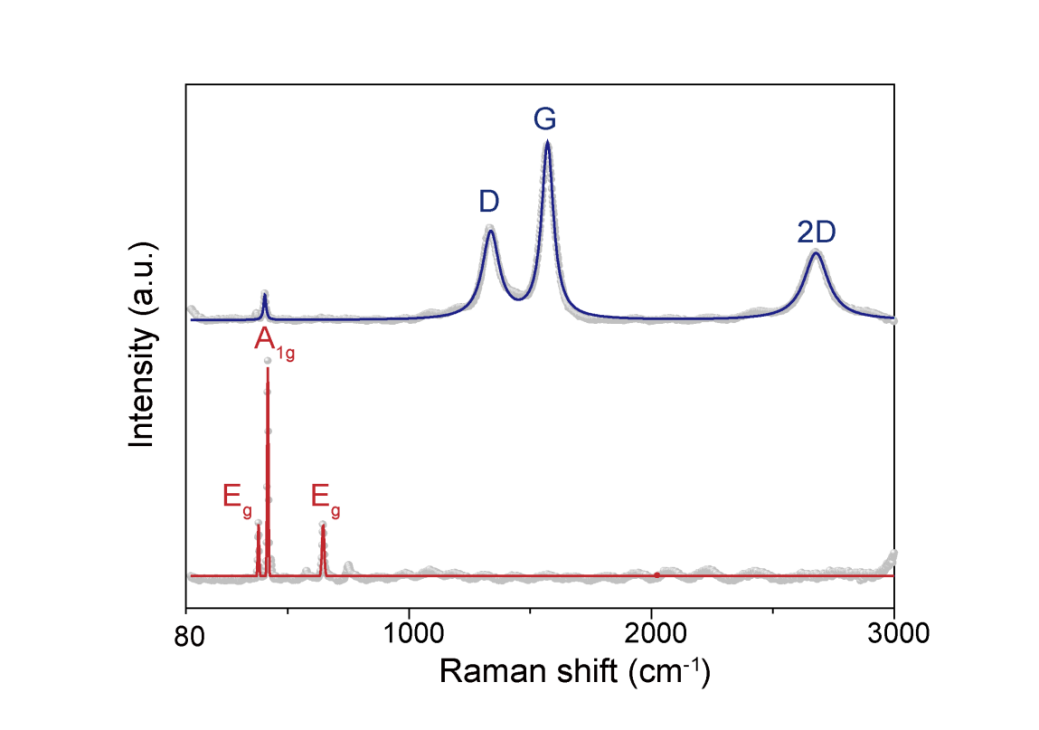


**Figure S12.** Representative Raman spectra of pristine Al_2_O_3_ powder (red) and Gr-skinned Al_2_O_3_ powder (blue).

**Figure S13. Raman spectra of Gr-skinned Al_2_O_3_ powder of different growth times and CH_4_/H_2_ ratios (the ratio of quantity flow of CH_4_ and H_2_)** **during growth procedure.**

The Raman spectra were collected with varying CH_4_/H_2_ ratios and growth times to determine the optimal growth conditions for producing Gr-skinned Al_2_O_3_ powders, as depicted in **Figure S13**. It is evident that a CH_4_/H_2_ ratio of 1:1 yields an average ID/IG value of approximately 0.55, which is lower than other growth conditions, indicating superior quality graphene skin obtained on Al_2_O_3_ powder.


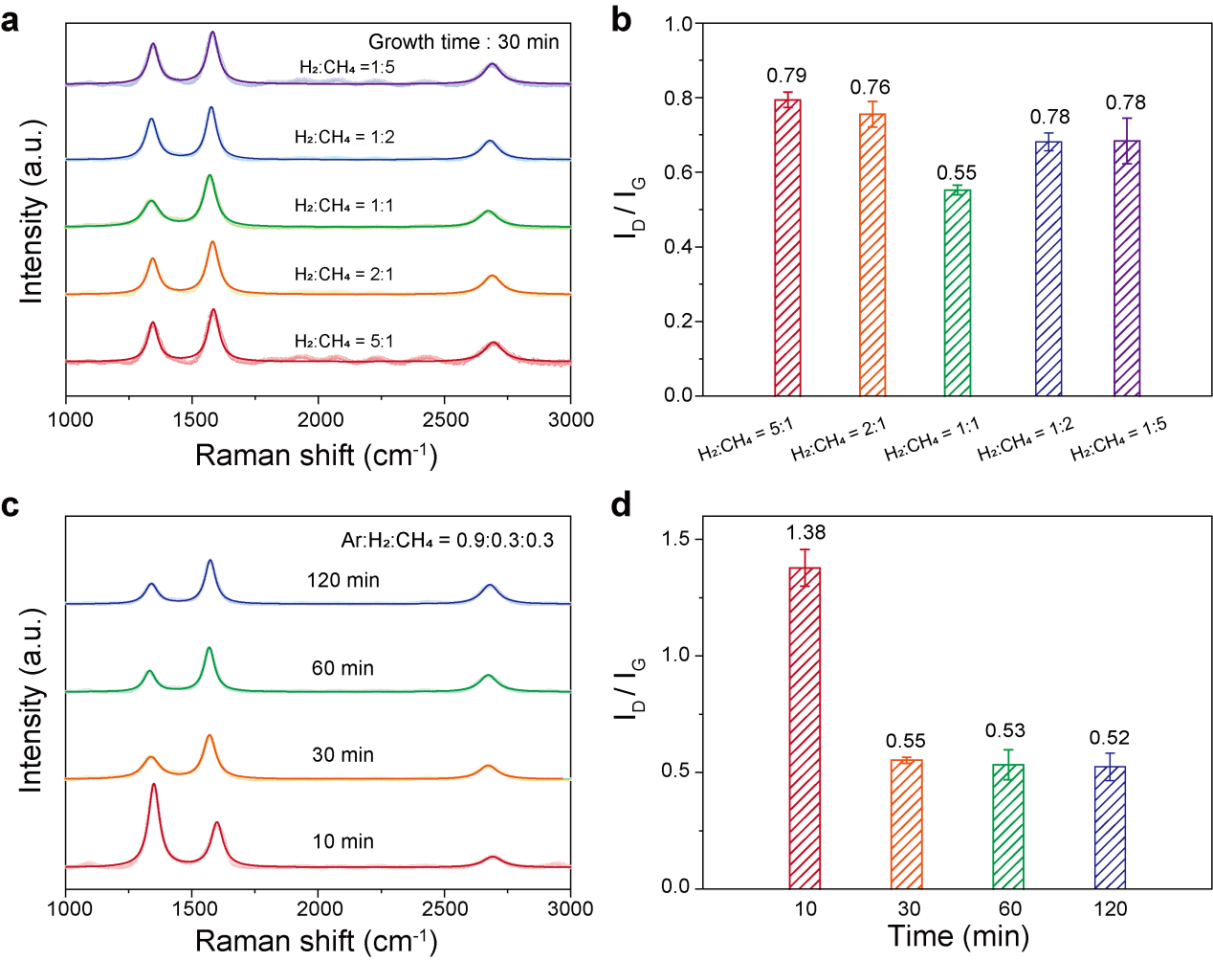


**Figure S13.** Raman spectra of Gr-skinned Al_2_O_3_ powder obtained under a) different growth times and c) CH_4_/H_2_ ratios during growth procedure along with the corresponding statistics of ID/IG values. b,d) All the spectra have been normalized to the G-peak intensity of each spectrum. The D-peak intensity decreases with elevating growth time, indicating an improvement in graphene quality.

**Figure S14. Batch-to-Batch Stability analysis of FB-CVD process.**


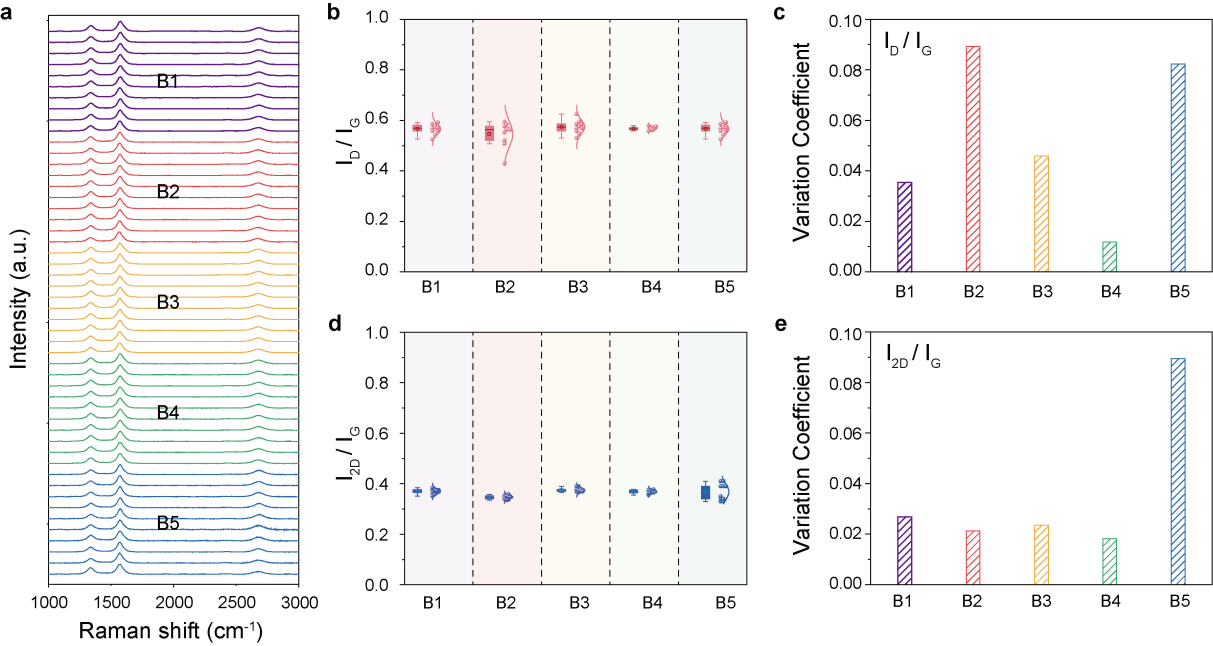


**Figure S14. Batch-to-Batch Stability analysis of FB-CVD process**. a) Raman spectra of 5 batch Gr-skinned Al_2_O_3_ powders (10 powders randomly-selected in each batch). b) I_D_/I_G_ statistics of Raman spectra in (a) and corresponding variation coefficient (c) of Gr-skinned Al_2_O_3_ powders in (b). d) I_2D_/I_G_ statistics of Raman spectra in (a) and corresponding variation coefficient (e) of Gr-skinned Al_2_O_3_ powders in (d). Growth conditions: 1100°C, atm, Ar/H_2_/CH_4_: 900/300/300 sccm, 30 min.

**Figure S15. X-ray diffraction (XRD) patterns of pristine α-Al_2_O_3_ and Gr-skinned Al_2_O_3_ powder at ~1100℃ of different growth times.**


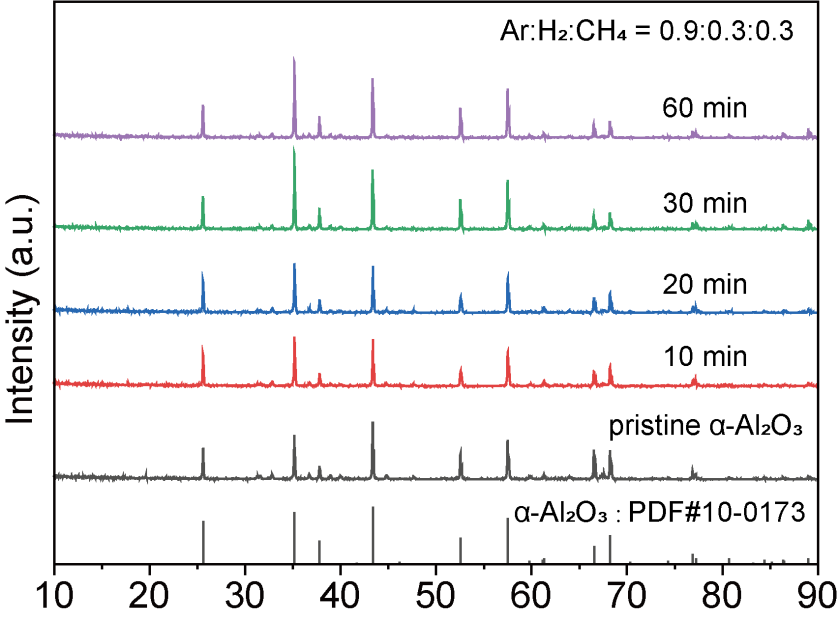


**Figure S15.** XRD patterns of pristine α-Al_2_O_3_ and Gr-skinned Al_2_O_3_ powder at ~1100℃ of different growth times.

**Figure S16. Training and convergence evaluation of neuroevolution machine learning potentials (NEP) for Al_2_O_3_.**

In recent years, machine learning (ML) has emerged as a powerful tool for predicting and uncovering hidden insights in materials. By incorporating extensive training data, machine-learning-trained potential functions have achieved accuracy close to quantum mechanics (Density Functional Theory, DFT) when predicting the energy and forces of molecular systems, significantly enhancing the reliability of molecular dynamics simulations.^[6]^ **Figure S16** illustrates the loss function of the neuroevolution potentials (NEP) trained for the Al_2_O_3_ system using the GPUMD package^[7]^ based on ML methods, where NEP exhibits good convergence. A comparison of the predicted energy, forces, and virial with the DFT reference values reveals that the NEP predictions are in close agreement with the DFT reference data. Therefore, NEP demonstrates an accuracy that is on par with DFT calculations. Subsequently, we applied the trained NEP for non-equilibrium molecular dynamics (NEMD) calculations of the thermal conductivity of Al_2_O_3_ (**Figure S17**). By fitting the thermal conductivity of Al_2_O_3_ with different sizes using the least squares method, we obtained a thermal conductivity of 40.16 W·m^−1^·K^−1^ for an infinitely large Al_2_O_3_ bulk, which is very close to the experimental results reported by Vera-Londono L and Paterson J.^[8-9]^ It is worth emphasizing that, compared to the sum of squared errors (SSE) for the EAM/alloy and ReaxFF potentials [SSE(EAM/alloy) = 1.3×10^-3^, SSE (ReaxFF) = 7.9×10^-5^], the SSE of the NEP is significantly lower, at 3.944×10^-7^. Therefore, the NEP yields lower fitting errors in the calculation results. Given the accuracy of the NEP method in calculating thermal conductivity, we further trained the NEP for the Gr-skinned Al_2_O_3_ powder (Gr/Al_2_O_3_ system). Similarly, we furnished the NEP training loss function along with the NEP-predicted energy, forces, and virial compared to the DFT data in **Figure S18**. The results indicate that the NEP has reliable computational accuracy for describing the Gr/Al_2_O_3_ system.


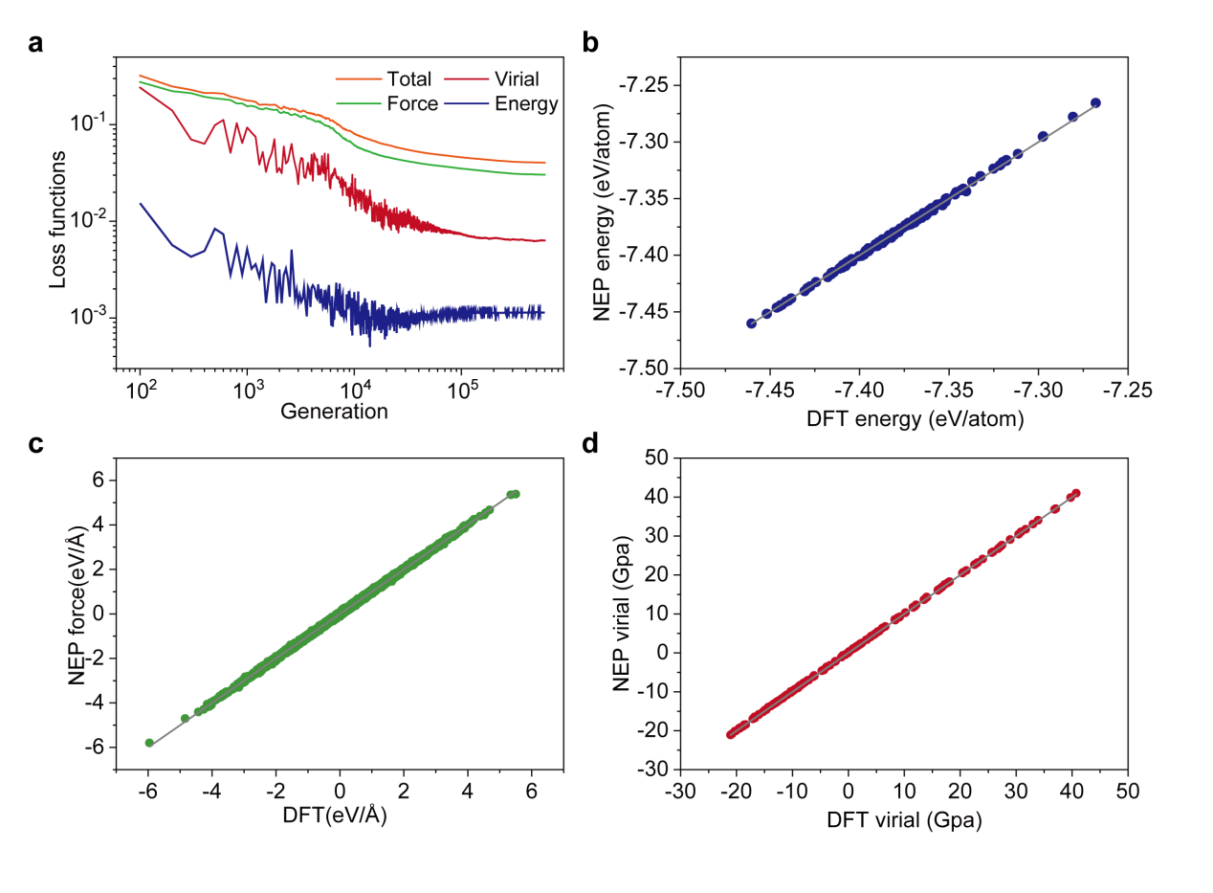


**Figure S16.** Training and convergence evaluation of NEP for Al_2_O_3_. a) Evolution of energy, force, and virial during the NEP training process. Comparison of the b) Energy, c) force, and d) virial obtained from NEP with the data calculated using density functional theory (DFT).

**Figure S17.** **Thermal conductivity of an Al_2_O_3_ block calculated using NEP.**


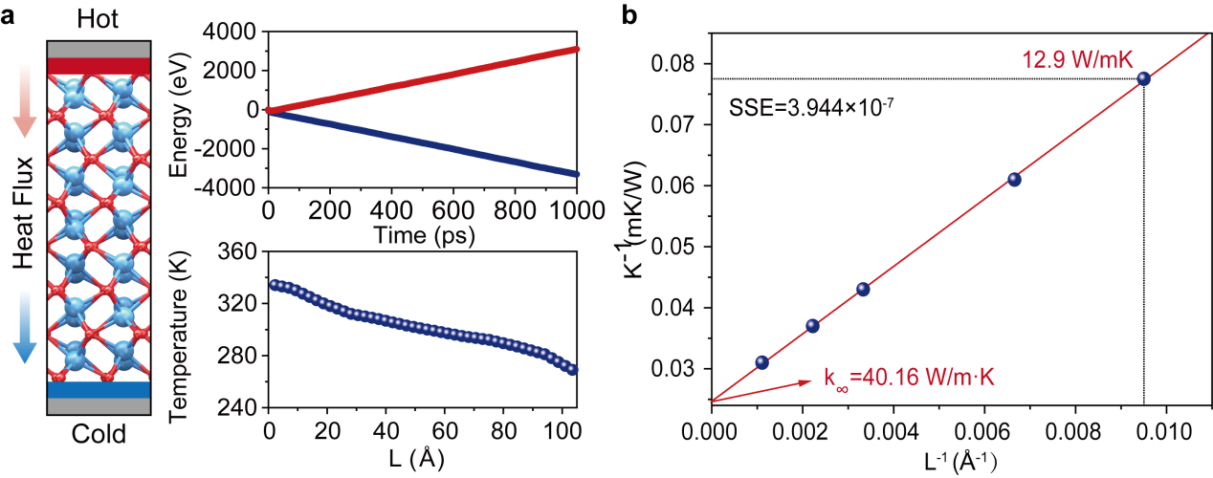


**Figure S17.** Thermal conductivity of an Al_2_O_3_ block calculated using NEP. a) Schematic modelling of Al_2_O_3_ block (left) in the NEP simulation and corresponding accumulated energy (heat sink, heat source) and temperature profile (right). b) Dependence of Al_2_O_3_ thermal conductivity on size, with the red line representing the least squares fit (SSE is 3.944×10^-7^) of the calculated data, and the intersection with the vertical axis gives a thermal conductivity of 40.16 W·m^-1^·K^-1^ for an infinite large Al_2_O_3_ block.

**Figure S18. Training and convergence evaluation of NEP for Gr-skinned Al_2_O_3_ powder.**


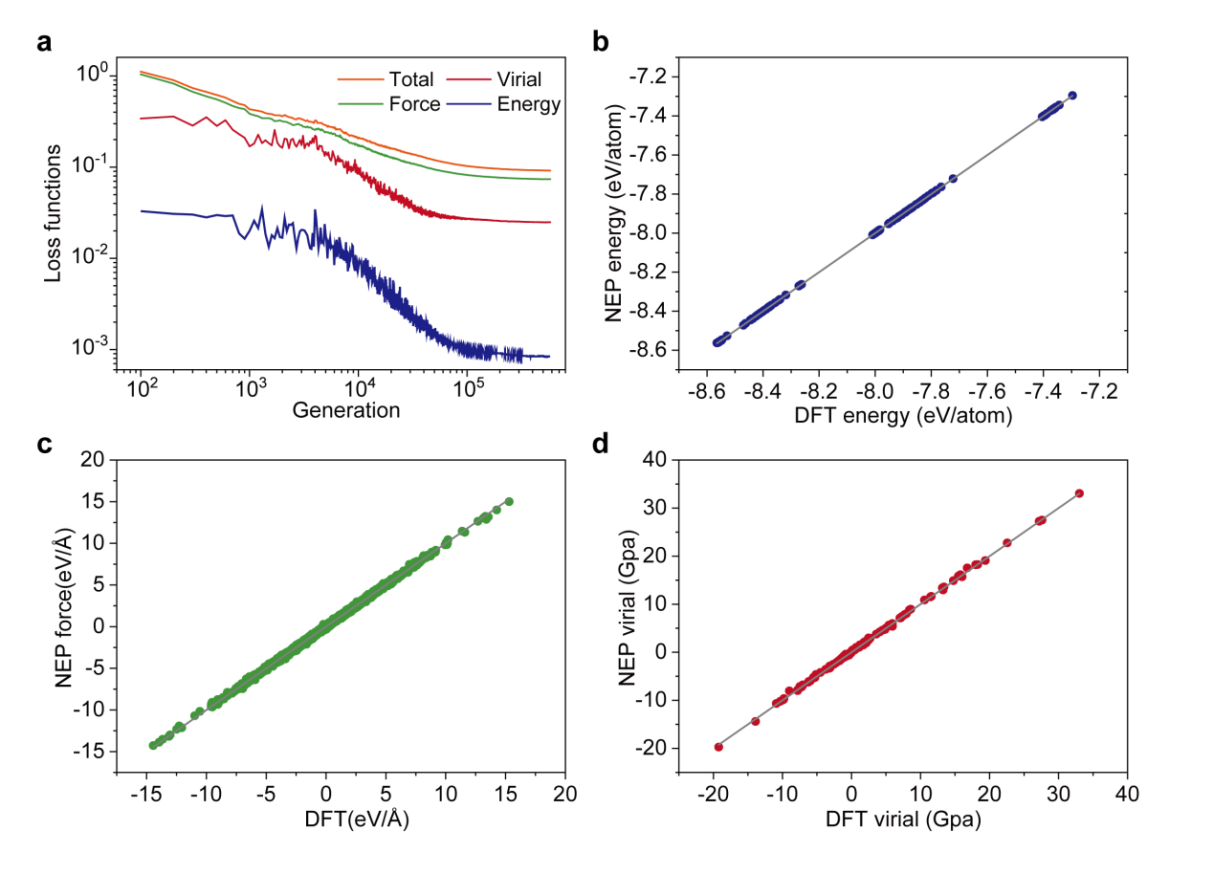


**Figure S18.** Training and convergence evaluation of NEP for Gr-skinned Al_2_O_3_ powder. a) Evolution of energy, force, and virial during the NEP training process. Comparison of the b) Energy, c) force, and d) virial obtained from NEP with data calculated from DFT.**Figure S19. Calculation model of the Gr-skinned Al_2_O_3_ powder.**

**
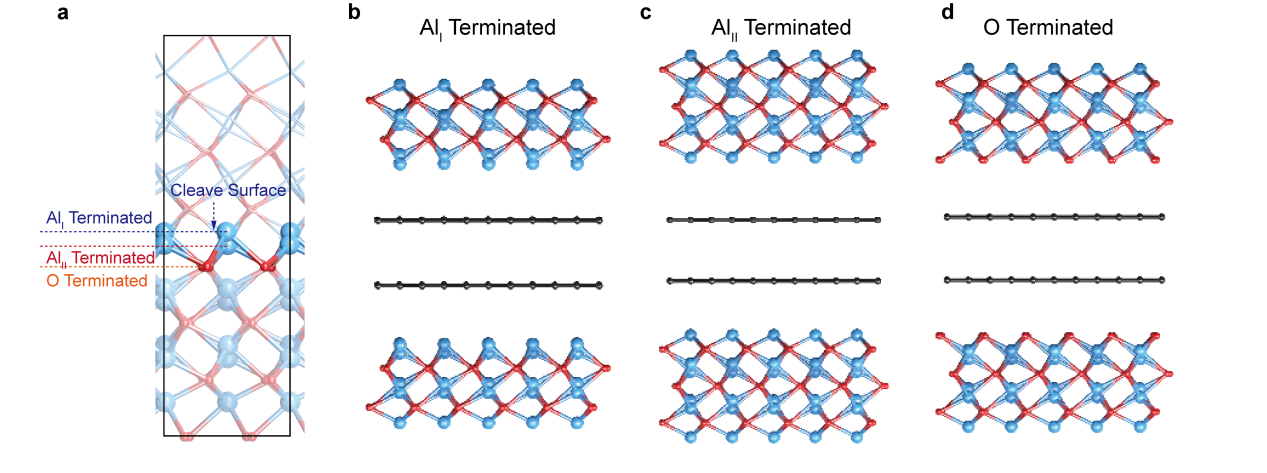
**

**Figure S19.** Calculation model of the Gr-skinned Al_2_O_3_ powder. a) Schematic of the atomic configurations for Al_2_O_3_ surfaces terminated with different elements. b-d) Stable configurations of Al_I_-, Al_II_-, and O-terminated surface structures.

**Figure S20. Density of phonon states (PDOS) at various contact interfaces in Gr-skinned Al_2_O_3_ powder.**


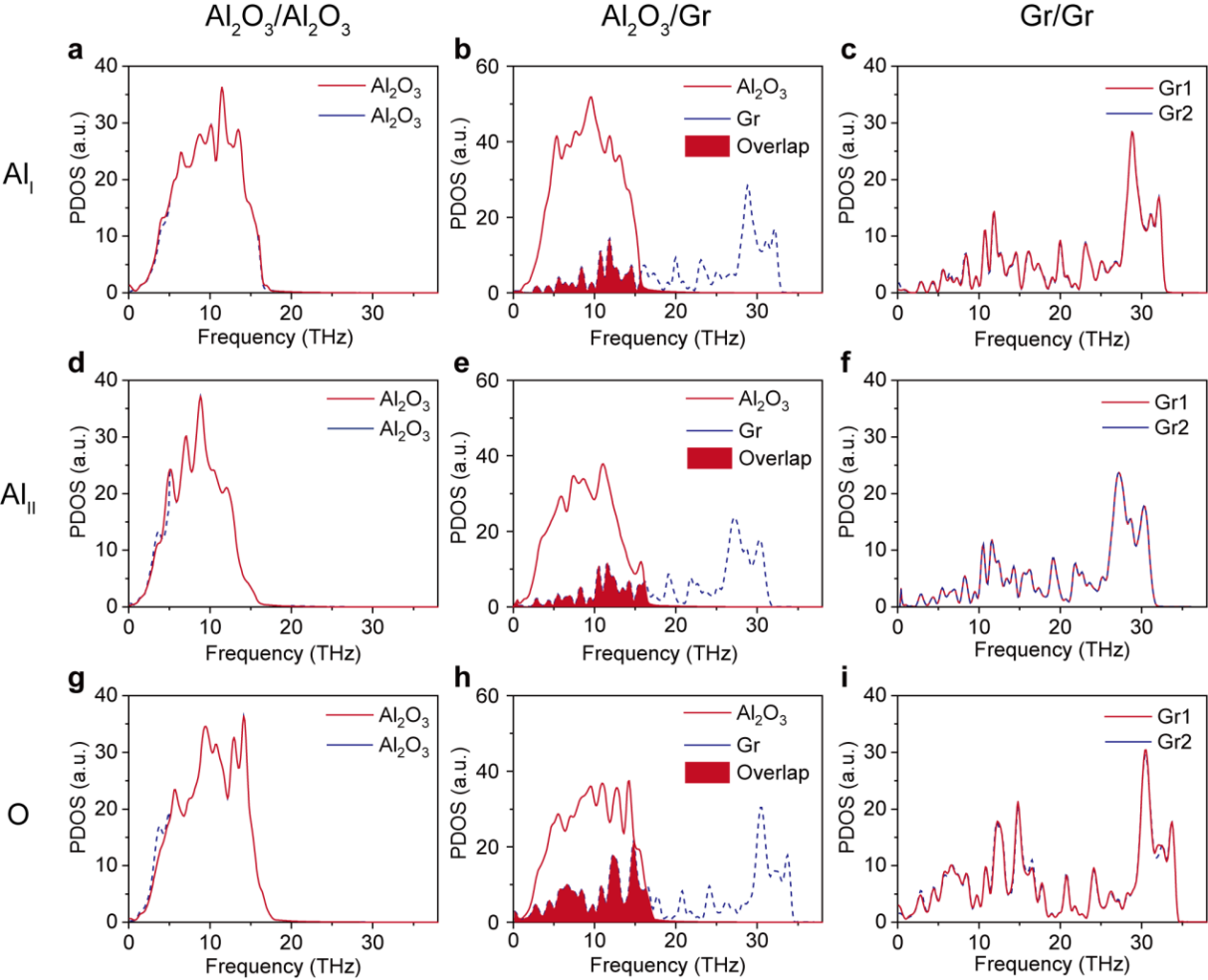


**Figure S20.** Density of phonon states (PDOS) at various contact interfaces in Gr-skinned Al_2_O_3_ powder, including the a-c) Al_I_ atomic contacts, d-f) Al_II_ atomic contacts, and g-i) O atomic contacts at the Al_2_O_3_/Al_2_O_3_, Al_2_O_3_/Gr and Gr/Gr interface, respectively.

**Figure S21. Schematic diagram of the Gr-skinned Al_2_O_3_ powder model.**

Computer simulation is a valuable engineering tool that can provide comprehensive thermal distribution and heat transfer data during the thermal conductivity process of composite materials. To further demonstrate the preferential thermal conductivity of graphene skin in the Gr-skinned Al_2_O_3_ powder system, we employed the commercial software ANSYS to calculate the temperature, heat flux, and heat flow of Gr-skinned Al_2_O_3_ powder. The material model and boundary conditions are illustrated in **Figure S21**, where the matrix powder is Al_2_O_3_ and the coating is graphene skin. The preparation of numerical grids, the establishment of CFD models, and all calculations were performed using the ICEM CFD and Fluent modules in ANSYS.

In the calculation, only the thermal conduction process was considered, with the effect of convective heat transfer being disregarded. The thermal analysis of Gr-skinned Al_2_O_3_ powder can be solved using only the energy conservation equation, which can be expressed as follows:

$\frac{\partial}{\partial t}\left( \rho h \right)=\nabla\cdot\left( k\nabla T \right)+S_{h}$,

where $\rho$ is the density, h the enthalpy, k the thermal conductivity, *T* the absolute temperature, *t* the time and *S_h_* the energy source. Under the condition of no internal heat source and steady-state heat conduction, the control equation is further simplified as:

$\nabla\cdot\left( k\nabla T \right)=0$,

Constant temperatures of 100℃ and 25℃ were applied to the hot and cold sides of the Gr-skinned Al_2_O_3_ powder respectively. The second-order upwind SIMPLE algorithm was employed to process the energy equation, with the relaxation factor maintained at the default setting and the relative residual of the energy equation less than 10^-7^ as the convergence criterion.

The material properties of Gr-skinned Al_2_O_3_ powder (i.e. density, heat capacity and thermal conductivity) have been set at constant values, listed in **Table S4**.


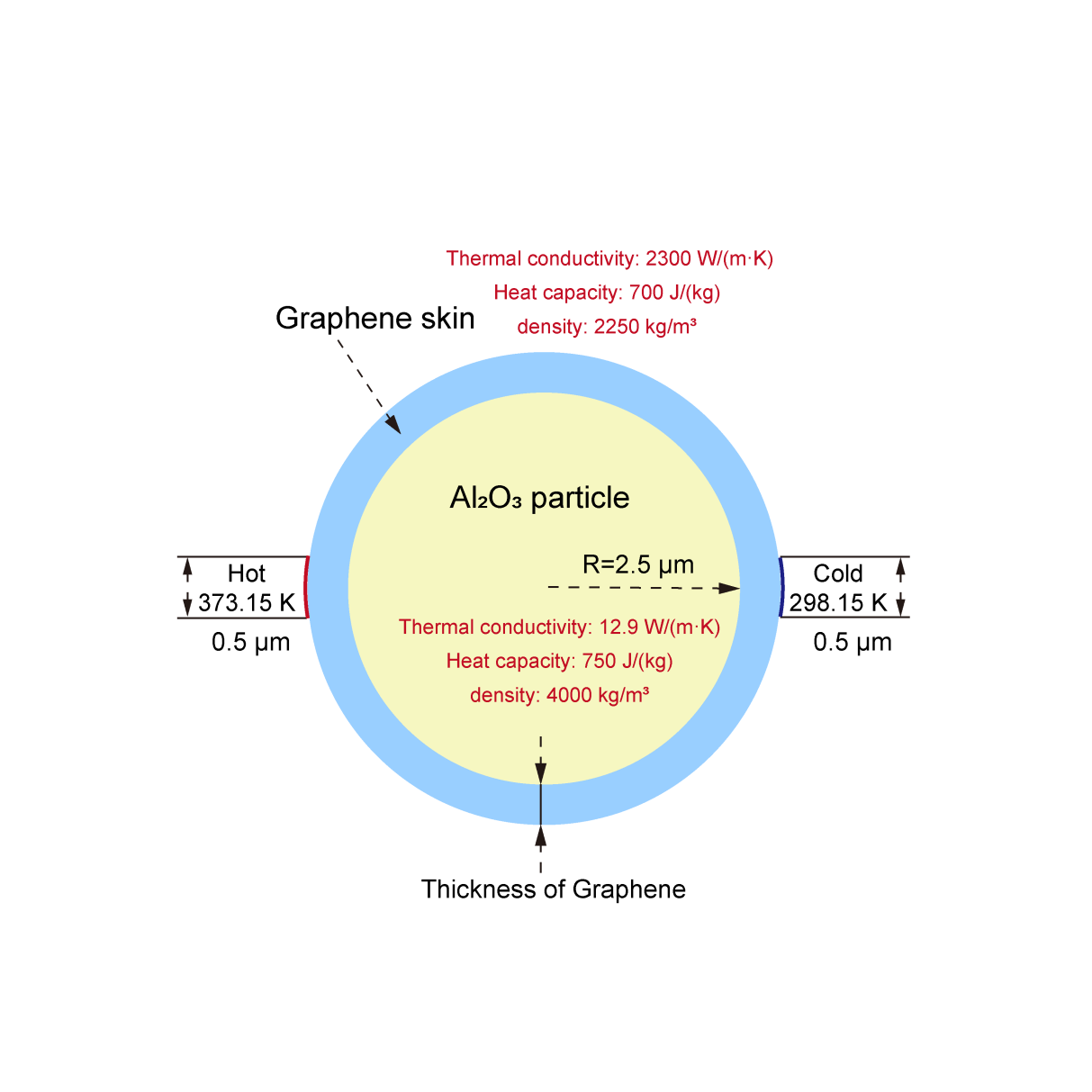


**Figure S21.** Schematic diagram of the Gr-skinned Al_2_O_3_ powder model.

**Figure S22. The relative change of heat flow varies with the grid size.**

Due to the spherical morphology of Gr-skinned Al_2_O_3_ powder, an O-type mesh is employed for grid partition. In order to minimize the impact of grid density on the numerical outcomes, the temperature of the Gr-skinned Al_2_O_3_ powder was simulated under operational conditions of varying grid sizes. The relative change rate of heat flow on the symmetrical section perpendicular to the line connecting the centre points of the hot and cold sides was calculated and shown in **Figure S21**. It can be seen that when the grid size is less than 6 nm, the heat flow tends to stabilize and remain basically unchanged when the temperatures of the hot and cold sides are fixed. In consideration of computational efficiency, the grid size for subsequent simulation calculations in this article was set at 6 nm.


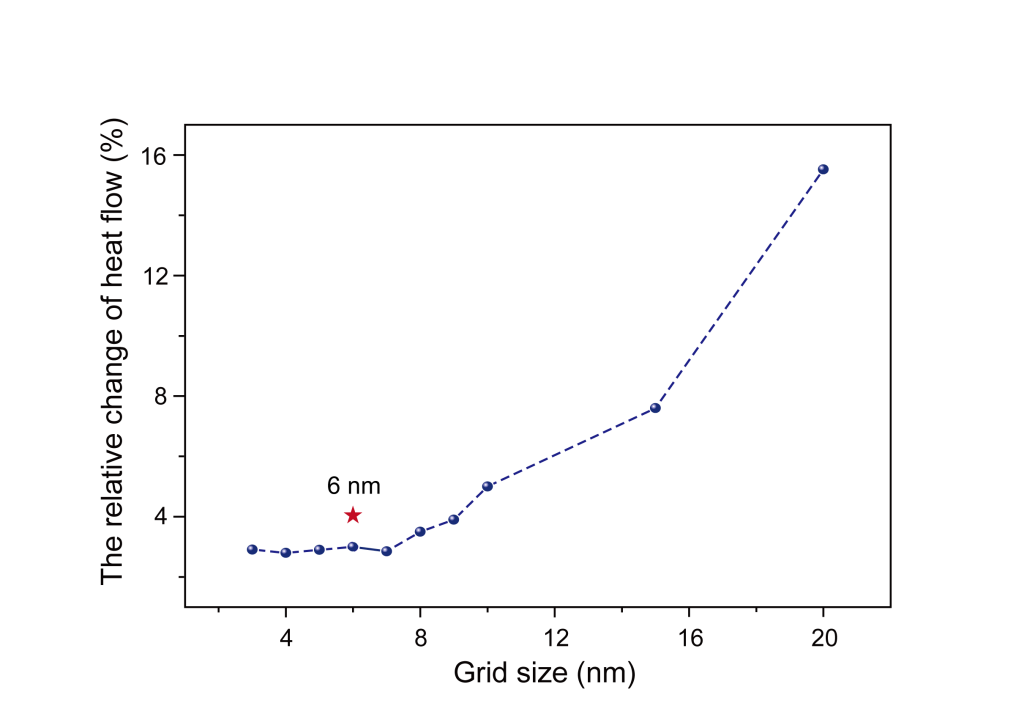


**Figure S22.** The relative change of heat flow varies with the grid size.

**Figure S23. Heat flux vector of Gr-skinned α-Al_2_O_3_ powder.**


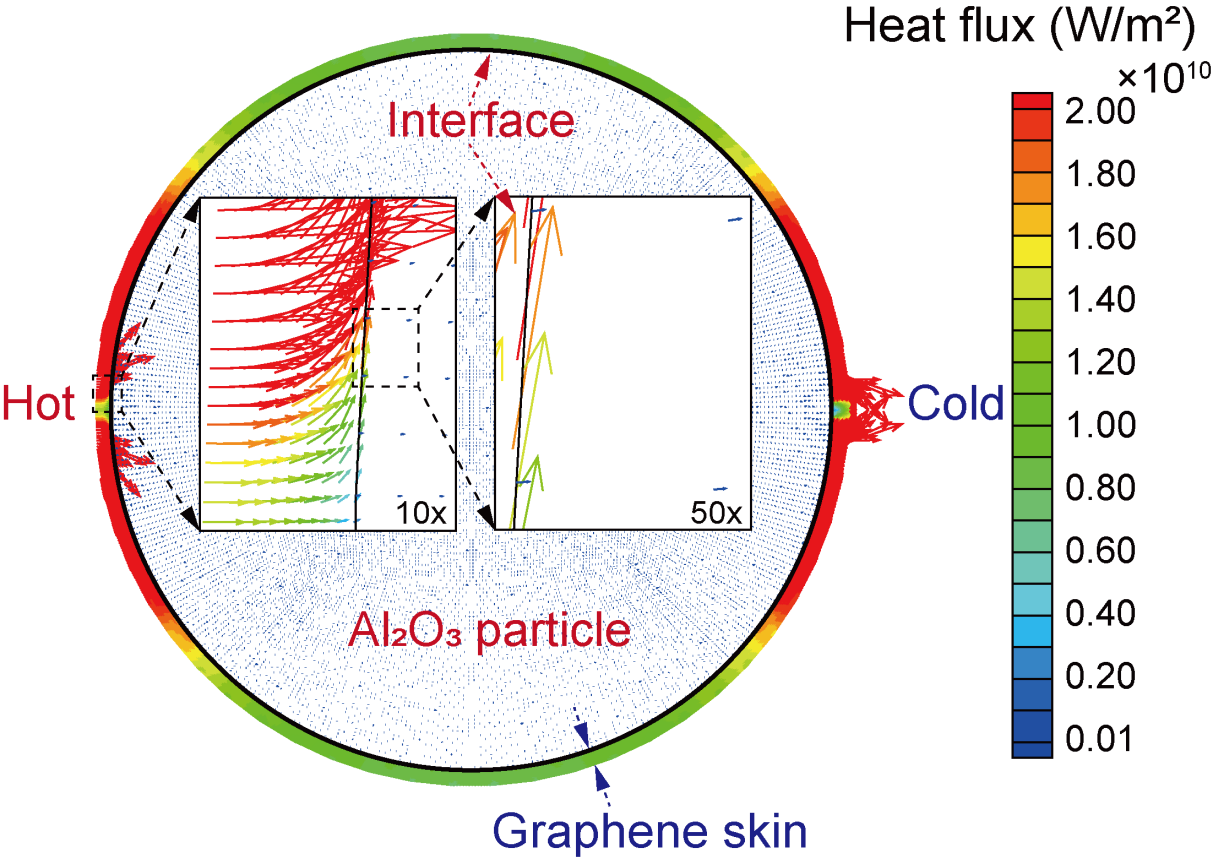


**Figure S23.** Heat flux vector of Gr-skinned α-Al_2_O_3_ powder (the graphene thickness was set at 100 nm).

**Figure S24.** **Heat flux vector of stacked Gr-skinned α-Al_2_O_3_ powders.**

**
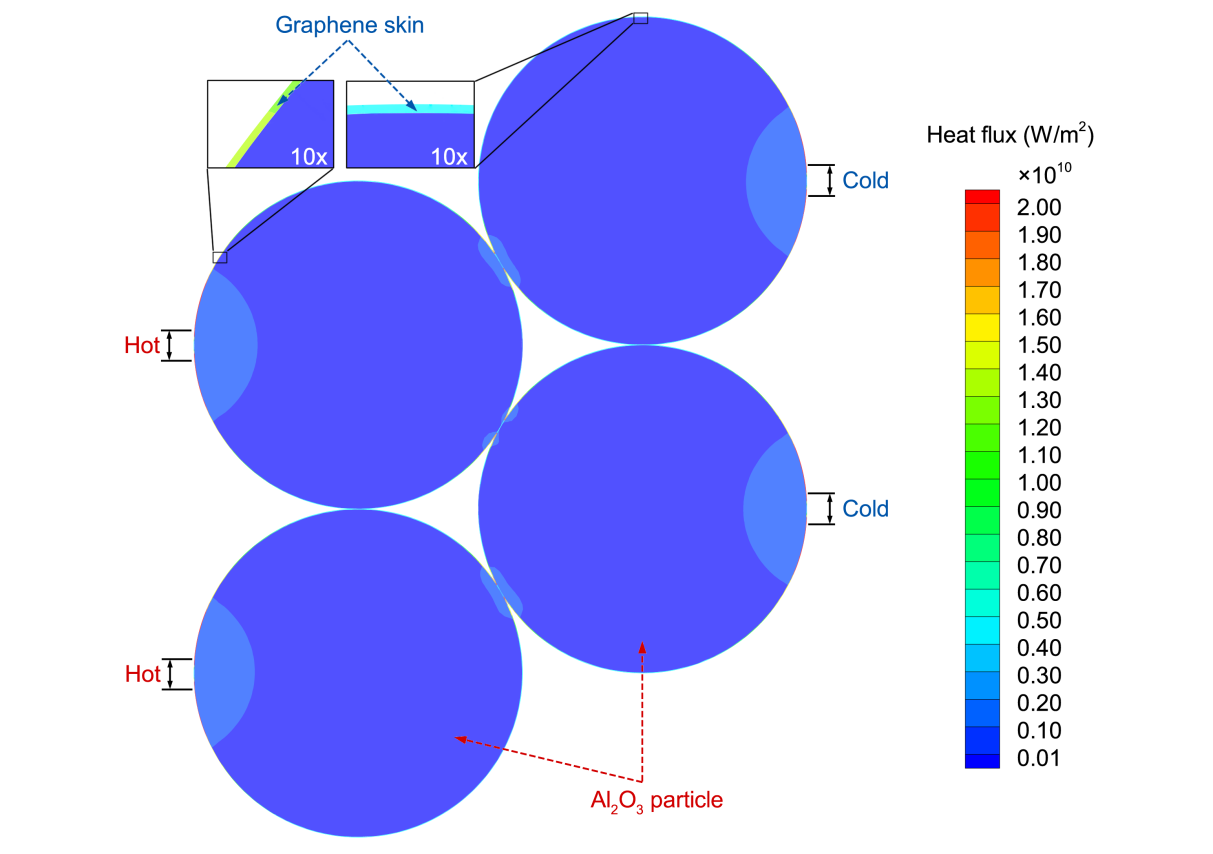
**

**Figure S24.** Heat flux vector of stacked Gr-skinned α-Al_2_O_3_ powders (the graphene thickness was set at 10.1 nm).

**Figure S25. Schematic of wettability of pristine Al_2_O_3_ powder and Gr-skinned Al_2_O_3_ powder.**


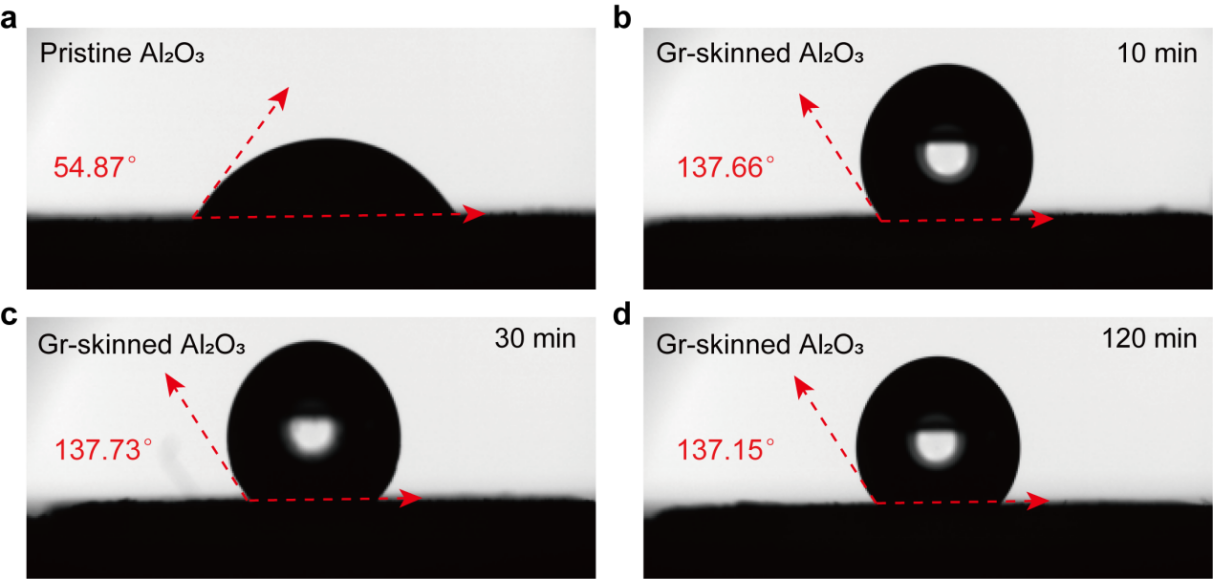


**Figure S25.** Schematic of wettability of pristine Al_2_O_3_ powder and Gr-skinned Al_2_O_3_ powder. The powder materials were compressed into tablets. The water contact angle (WCA) of a) pristine Al_2_O_3_ powder materials and Gr-skinned Al_2_O_3_ powder of different growth times. b) 10 min c) 30 min d) 120 min.

**Figure S26. Brunner-Emmet-Teller (BET) surface area measurements of pristine Al_2_O_3_ powder and Gr-skinned Al_2_O_3_ powder materials of different particle sizes.**

**
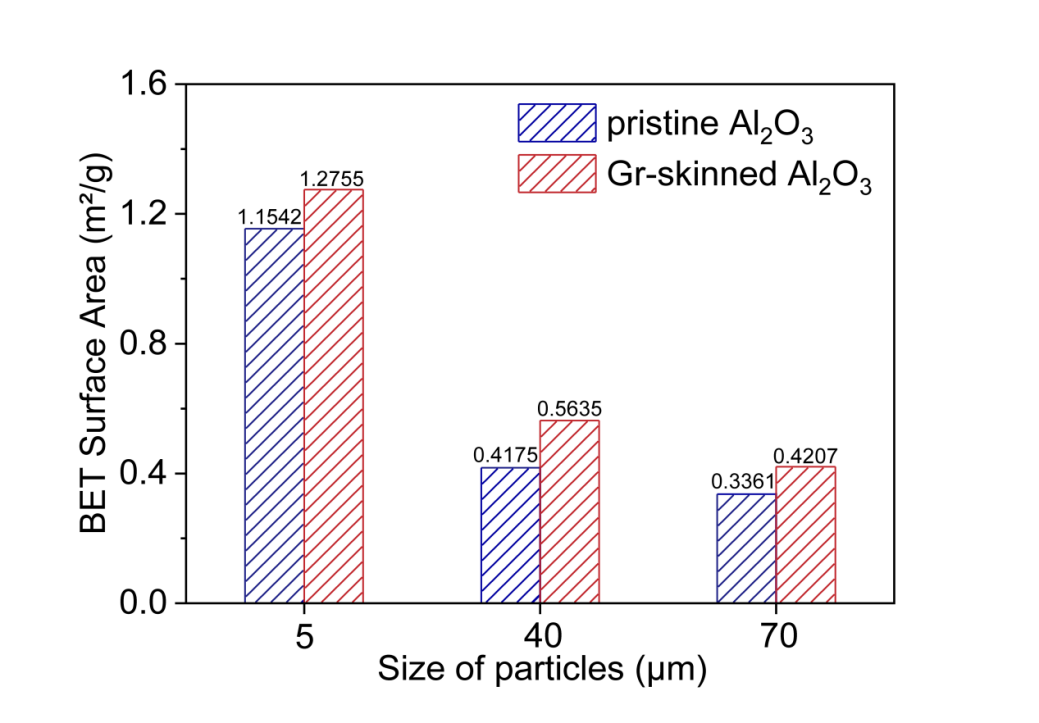
**

**Figure S26.** BET surface area of pristine Al_2_O_3_ powder and Gr-skinned Al_2_O_3_ powder materials of different particle sizes (5/40/70 μm).

**Figure S27. Photographs of the Gr-skinned Al_2_O_3_ powder** **based thermal interface material (TIM).**


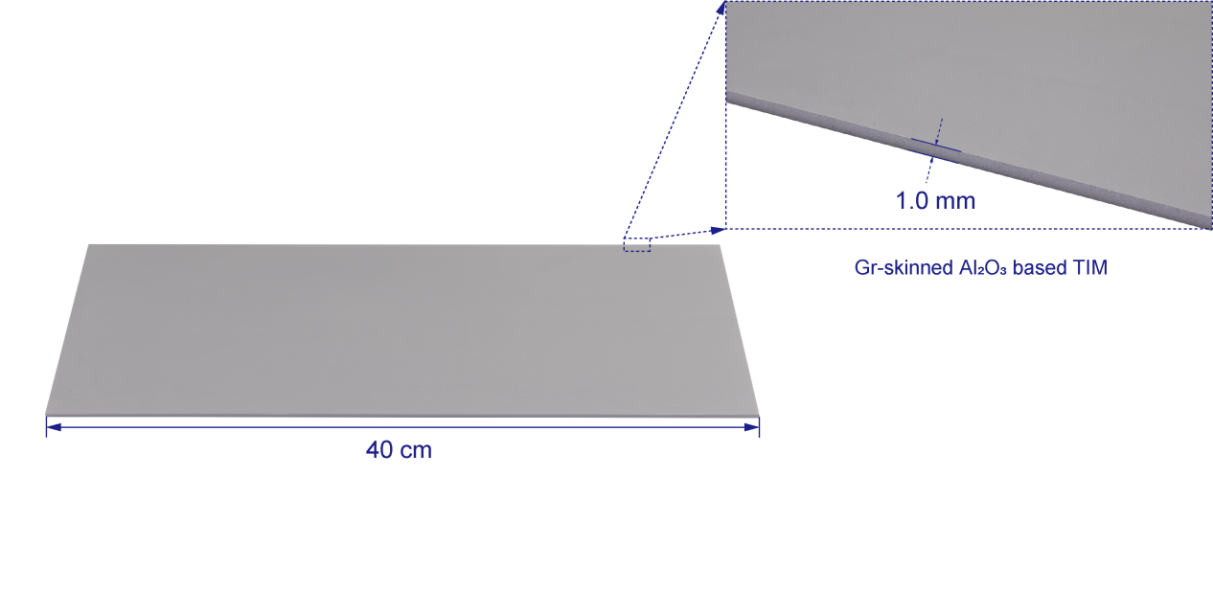


**Figure S27.** Photographs of the Gr-skinned Al_2_O_3_ based TIM. The dimensions of the TIM were 20 × 40 cm and its thickness was 1.0 mm. This can be readily processed to the requisite dimensions for the intended practical application.

**Figure S28. Illustration o****f Hot Disk measurements to detect the thermal conductivity of** **pristine Al_2_O_3_ powder and Gr-skinned Al_2_O_3_ powder.**

The apparatus is designed for precision analysis of the thermal transport properties of a given substance, including thermal conductivity, thermal diffusivity, and specific heat capacity. A transient thermal pulse generated by a double helix metal foil probe is employed to ascertain the thermal conductivity of a sample by measuring the temperature change of the probe over time. The principal experimental setup for the Hot Disk measurement comprises a double helix structure probe that is capable of both heating and temperature sensing. This configuration is suitable for a wide range of materials with varying geometries and dimensions, including solids, pastes, and liquids, while adhering to the ISO Standard 22007-2. During the measurement process, the probe was fully inserted into the sample, which was compressed into tablets, to ensure comprehensive coverage and optimal thermal contact. Once the heating process is initiated, temperature fluctuations are monitored, enabling precise determination of thermal conductivity.


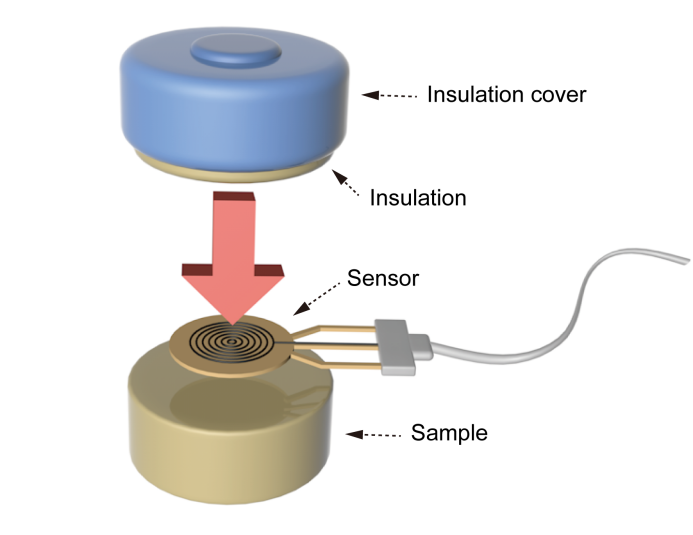


**Figure S28.** Illustration of Hot Disk measurements based on transient plane source method to detect the thermal conductivity of pristine Al_2_O_3_ powder and Gr-skinned Al_2_O_3_ powder.

**Figure S29. Photograph of the experimental platform for the testing progress of the Gr-skinned Al_2_O_3_ powder based TIM sandwiched between a light-emitting diode (LED) lamp and a heat sink.**

The Gr-skinned Al_2_O_3_ powder based TIM, as illustrated in **Figure S24**, was cut into corresponding dimensions to fit the gap between the LED lamp and a heat sink to simulate the heat dissipation process. An infrared camera was set up to detect the temperature of the LED lamp to validate the performance of the TIM for thermal management in modern electronics.

**
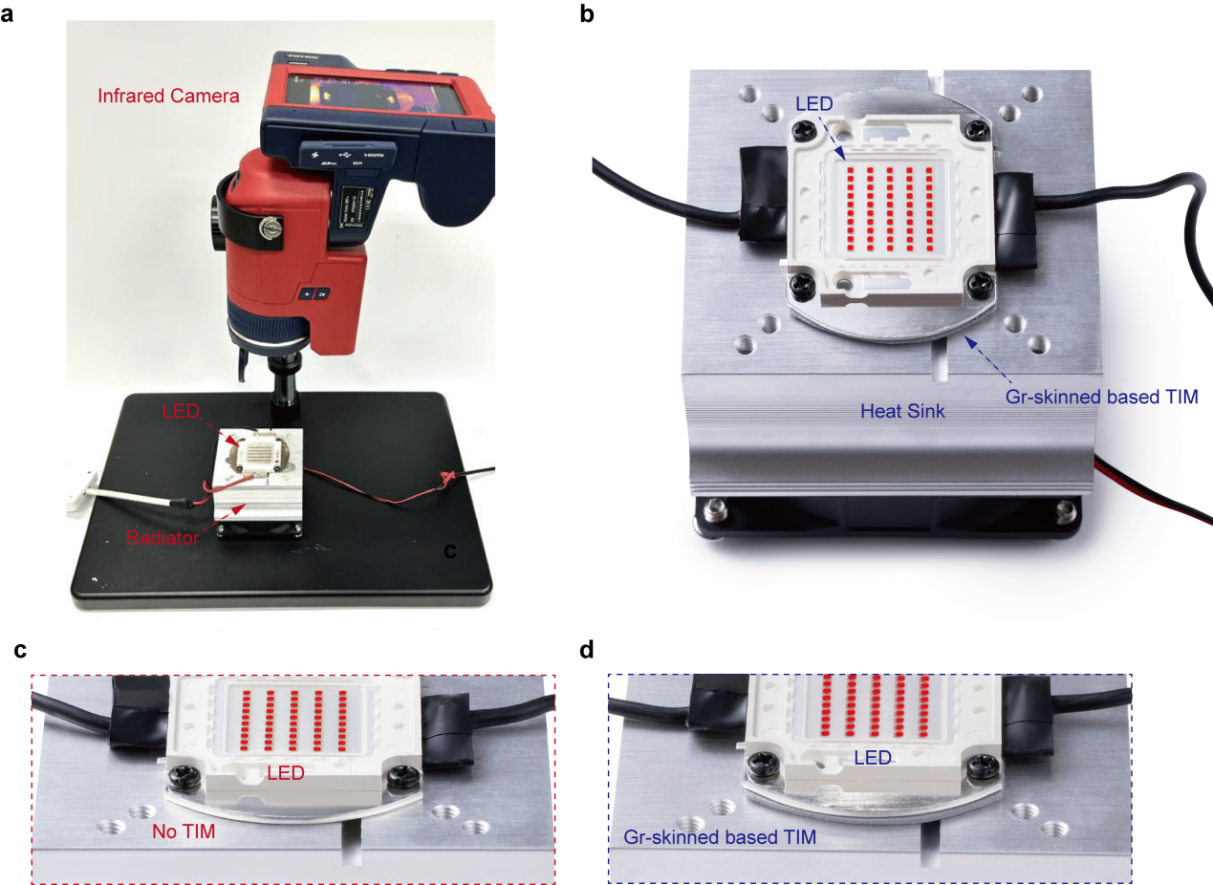
**

**Figure S29.** Photographs of the experimental platform for the testing progress of the Gr-skinned Al_2_O_3_ based TIM sandwiched between the LED lamp and a heat sink. a) Photograph of the experimental platform. b) Morphology of the LED and radiator. The magnified photographs of the interface between the LED and radiator with c) no TIM and d) Gr-skinned Al_2_O_3_ based TIM.

**Figure S30. Temperature cloud diagrams of the LED during the cooling process.**


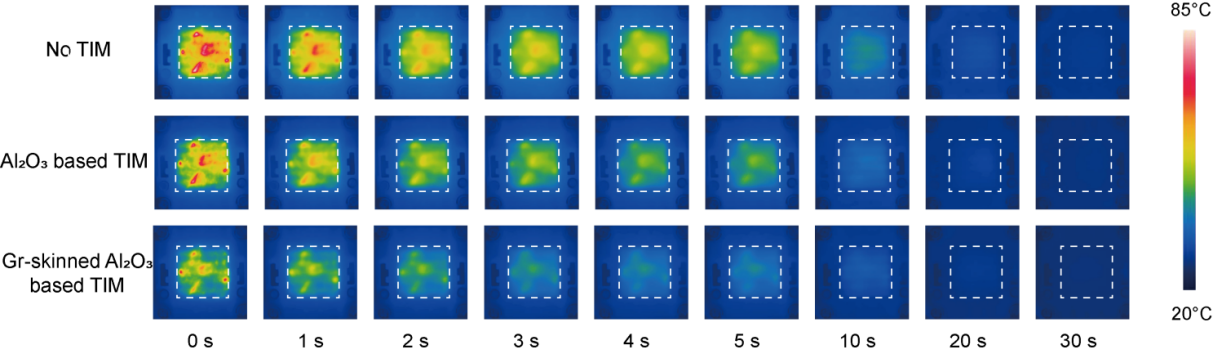


**Figure S30.** Temperature cloud diagrams of the LED during the cooling process when using no TIM, Al_2_O_3_ based TIM and Gr-skinned Al_2_O_3_ based TIM.

**Figure S31. Thermal stability of Gr-skinned Al₂O₃ powder based TIM.**


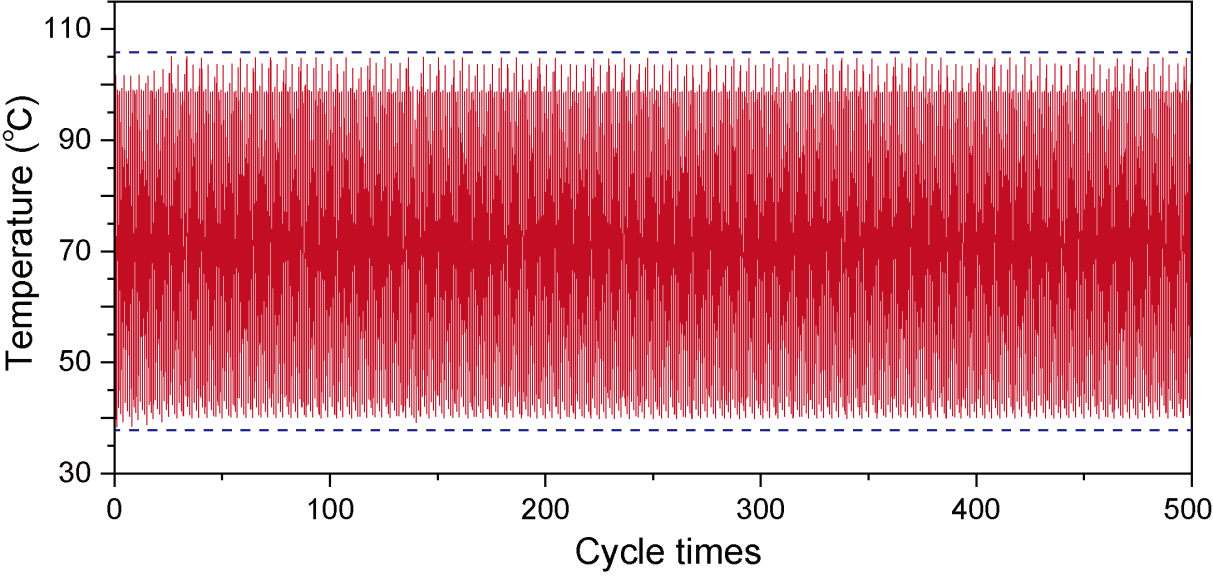


**Figure S31.** Thermal stability of Gr-skinned Al₂O₃ powder based TIM under high and low temperature cycling tests in 500 cycles.

**Table S1. Grid quality of fluidized bed model.**

| Mesh Metric | Skewness |
| --- | --- |
| Min | 1.3057 e-10 |
| Max | 0.657 |
| Average | 1.0428 e-2 |
| Standard Deviation | 3.8098 e-2 |

**Table S2. Statistics of *I_D_/I_G_* and *I_2D_/I_G_* (intensity ratio of 2D and G peaks) in Raman spectra in Figure 2g.**

The statistics of *I_D_/I_G_* and *I_2D_/I_G_* in **Table S2** were collected from 12 randomly selected Gr-skinned Al_2_O_3_ powders. The samples were numbered 1-12 from bottom to top in the illustration of **Figure 2g** in the main text. It can be seen that the values of *I_D_/I_G_* and *I_2D_/I_G_* are very uniform, further confirming the excellent uniformity of graphene on the Al_2_O_3_ powders.

|  | **1** | **2** | **3** | **4** | **5** | **6** | **7** | **8** | **9** | **10** | **11** | **12** |
| --- | --- | --- | --- | --- | --- | --- | --- | --- | --- | --- | --- | --- |
| ***I*_d_*/I*_g_** | 0.56 | 0.56 | 0.56 | 0.47 | 0.51 | 0.56 | 0.5 | 0.46 | 0.46 | 0.46 | 0.47 | 0.46 |
| ***I*_2d_*/I*_g_** | 0.33 | 0.33 | 0.33 | 0.33 | 0.34 | 0.39 | 0.41 | 0.41 | 0.39 | 0.35 | 0.39 | 0.34 |

**Table S3. Summary of the thermal conductivity of Al_2_O_3_ at various sizes, as determined using the EAM/alloy and ReaxFF potentials.**

According to the extant literature, the most commonly used potential functions for describing Al_2_O_3_ in molecular dynamics simulations are the EAM/alloy potential^[10]^ and the ReaxFF potential.^[11]^ Preliminary calculations of the thermal conductivity of Al_2_O_3_ were performed using these two potentials. Depending on the differences in the computational models, the thermal conductivity values ranged from 0.714-3.73 W·m^−1^·K^−1^ for EAM/alloy potential and 4.58-20.41 W·m^−1^·K^−1^ for the ReaxFF potential, respectively (**Table S3**). To mitigate the effects of size dependency, we fitted the thermal conductivity of an infinitely large Al_2_O_3_ bulk using the least squares method, yielding values of 4.00 W·m^−1^·K^−1^ and 30.89 W·m^−1^·K^−1^, which are significantly lower than the experimentally reported thermal conductivity values of pure-phase Al_2_O_3_ materials.^[12]^ This discrepancy is primarily due to the inability of the EAM/alloy potential and ReaxFF potential to accurately describe the atomic bonding in Al_2_O_3_ (including the complex hybrid characteristics of ionic and covalent bonds), as well as their limitations in accurately describing phonon transport and phonon scattering mechanisms, leading to varying degrees of underestimation in the description of thermal conductivity.^[13-14]^

| **L (Å)** | 100 | 150 | 300 | 450 | 900 | ∞ |
| --- | --- | --- | --- | --- | --- | --- |
| **k_(EAM/alloy)_ [W·m^-1^·K^-1^]^a)^** | 0.714 | 0.91 | 1.35 | 1.92 | 3.73 | 4 |
| **k_(ReaxFF)_ [W·m^-1^·K^-1^]^b)^** | 4.58 | 6.25 | 11.76 | 13.3 | 20.41 | 30.89 |

^a)^ EAM/alloy and ^b)^ReaxFF potentials are referenced from the works of Hong, Streitz et al.^[10-11]^ Here, ∞ represents the infinite size of Al_2_O_3_, determined from a least squares fit. The sum of squared errors (SSE) for the least squares fit of EAM/alloy potential is 1.3×10^-3^, and the SSE for the ReaxFF potential is 7.9×10^-5^.

**Table S4. Thermal properties of Gr-skinned Al_2_O_3_ powders.**

| **Part** | Density  [kg/m^3^] | Heat Capacity  [J/(kg·K)] | Thermal Conductivity  [W·m^-1^·K^-1^] |
| --- | --- | --- | --- |
| **Graphene** | 2250 | 700 | 2300 |
| **Al_2_O_3_** | 4000 | 750 | 12.9 |

**Table S5. Comparison of Gr-skinned Al_2_O_3_ powder materials based TIM and other thermal conductive fillers based TIMs.^[15-24]^**

| **Filler/Matrix** | **Methods** | **Loading [vol%]** | **Thermal**  **diffusivity**  **[mm^2^·s^-1^]** | **Electrical resistivity [Ω·cm]** | **Thermal conductivity**  **[W·m^-1^·K^-1^]** | **Year** |
| --- | --- | --- | --- | --- | --- | --- |
| 80% 30 μm and 20% 5 μm Al_2_O_3_/epoxy | blending | 50% | - | - | 1.364 | 2019^[15]^ |
| Core-shell Cu@Al_2_O_3_/epoxy | solution-phase synthesis | 10% | - | 2.3×10^13^ | 1.32 | 2022^[16]^ |
| Al_2_O_3_-PCPA-Si69/epoxy natural rubber | modification | 30% | - | - | 0.3773 | 2022^[17]^ |
| 20 vol% branched Al_2_O_3_ and  0.5 wt% CNTs  /silicon rubber | modification | 20% | - | - | 1.307 | 2023^[18]^ |
| Al_2_O_3_/epoxy | blending | 50 wt.% | - | - | 0.46 | 2015^[19]^ |
| Al_2_O_3_@POSS  /rubber | casting | 20% | 0.16 | - | 0.28 | 2023^[20]^ |
| 60% 45 μm Al_2_O_3_ and 40% 3 μm Al_2_O_3_/epoxy | modification | 80 wt.% | - | - | 2.47 | 2023^[21]^ |
| 5 μm G-Al_2_O_3_  /SA^a)^ | deposition | 66% | - | - | 1.65 | 2014^[22]^ |
| 4 μm Al_2_O_3_  /epoxy | blending | 34.7% | - | - | 0.82 | 2019^[23]^ |
| 2 μm Al_2_O_3_  /silicone rubber | modification | 56.9% | - | - | 1.73 | 2020^[24]^ |
| **Gr-skinned Al_2_O_3_**  **/vinyl silicone** | **FBCVD** | **95.3 wt.%** |  |  | **6.44** | **This work** |

^a)^ G is the abbreviation of graphene.

References:

[1] D. Geldart, Types of gas fluidization, *Powder Technol.* **1973**, *7*, 285.

[2] E. Peirano, V. Delloume, B. Leckner, Two- or three-dimensional simulations of turbulent gas–solid flows applied to fluidization, *Chem. Eng. Sci.* **2001**, *56*, 4787.

[3] A. Klimanek, W. Adamczyk, S. Kallio, P. Kozołub, G. Węcel, A. Szlęk, Experimental and numerical study of pseudo-2D circulating fluidized beds, *Particuology* **2016**, *29*, 48.

[4] D. Gidaspow, Hydrodynamics of Fiuidizatlon and Heat Transfer: Supercomputer Modeling, *Appl. Mech. Rev.* **1986**, *39*, 1.

[5] J. Ding, D. Gidaspow, A bubbling fluidization model using kinetic theory of granular flow, *AIChE J.* **1990**, *36*, 523.

[6] Z. Y. Fan, Y. Z. Wang, P. H. Ying, K. K. Song, J. J. Wang, Y. Wang, Z. Z. Zeng, X. Ke, E. Lindgren, J. M. Rahm, A. J. Gabourie, J. H. Liu, H. K. Dong, J. Y. Wu, C. Yue, Z. Zheng, S. Jian, P. Erhart, Y. J. Su, T. Ala-Nissila, GPUMD: A package for constructing accurate machine-learned potentials and performing highly efficient atomistic simulationsm, *J. Chem. Phys.* **2022**, *157*, 114801.

[7] L. Vera-Londono, A. Ruiz-Clavijo, O. Caballero-Calero, M. Martín-González, Understanding the thermal conductivity variations in nanoporous anodic aluminum oxide, *Nanoscale Adv.* **2020**, *2*, 4591.

[8] J. Paterson, D. Singhal, D. Tainoff, J. Richard, O. Bourgeois, Thermal conductivity and thermal boundary resistance of amorphous Al_2_O_3_ thin films on germanium and sapphire, *J. Appl. Phys.* **2020**, *127*, 12.

[9] Z. Y. Fan, Z. Z. Zeng, C. Z. Zhang, Y. Z. Wang, K. K. Song, H. K. Dong, Y. Chen, T. A. Nissila, Neuroevolution machine learning potentials: Combining high accuracy and low cost in atomistic simulations and application to heat transport, *Phys. Rev. B* **2021**, *104*, 104309.

[10] S. Hong, A. C. T. van Duin, Atomistic-Scale Analysis of Carbon Coating and Its Effect on the Oxidation of Aluminum Nanoparticles by ReaxFF-Molecular Dynamics Simulations, *J. Phys. Chem. C* **2016**, *120*, 9464.

[11] F. H. Streitz, J. W. Mintmire, Electrostatic potentials for metal-oxide surfaces and interfaces, *Phys. Rev. B* **1994**, *50*, 11996.

[12] P. K. Schelling, S. R. Phillpot, P. Keblinski, Comparison of atomic-level simulation methods for computing thermal conductivity, *Phys. Rev. B* **2002**, *65*, 144306.

[13] B. J. Lee, J. H. Shim, M. I. Baskes, Semiempirical atomic potentials for the fcc metals Cu, Ag, Au, Ni, Pd, Pt, Al, and Pb based on first and second nearest-neighbor modified embedded atom method, *Phys. Rev. B* **2003**, *68,* 144112.

[14] G. P. P. Pun, V. Yamakov, J. Hickman, E. H. Glaessgen, Y. Mishin, Development of a general-purpose machine-learning interatomic potential for aluminum by the physically informed neural network method, *Phys. Rev. Mater.* **2020**, *4*, 113807.

[15] C. Chen, Y. Xue, X. W. Li, Y. F. Wen, J. W. Liu, Z. G. Xue, D. A. Shi, X. P. Zhou, X. L. Xie, Y. W. Mai, High-performance epoxy/binary spherical alumina composite as underfill material for electronic packaging, *Compos. Part A-Appl. Sci. Manuf.* **2019**, *118*, 67.

[16] Z. L. Wang, Y. A. Zhang, J. A. Yi, N. Cai, J. Guo, Core-shell Cu@Al_2_O_3_ fillers for enhancing thermal conductivity and retaining electrical insulation of epoxy composites, *J. Alloy. Compd.* **2022**, *928*, 167123.

[17] X. Xie, D. Yang, Multi-functionalization of Al_2_O_3_ nanoparticles for enhancing thermal conductivity of epoxy natural rubber composites, *Appl. Surf. Sci.* **2022**, *602*, 154335.

[18] Y. G. Ouyang, Z. X. Yang, T. Y. Long, H. F. Tian, L. Y. Bai, X. F. Li, F. L. Yuan, Preparation of branched Al_2_O_3_ and its synergistic effect with carbon nanotubes on the enhancement of thermal conductive and electrical insulation properties of silicone rubber composites, *Mater. Today Commun.* **2023**, *34*, 105239.

[19] Z. F. Gao, L. Zhao, Effect of nano-fillers on the thermal conductivity of epoxy composites with micro-Al_2_O_3_ particles, *Mater. Des.* **2015**, *66*, 176.

[20] L. Mirizzi, M. D'Arienzo, R. Nisticò, G. Fredi, S. Diré, E. Callone, A. Dorigato, L. Giannini, S. Guerra, S. Mostoni, B. Di Credico, R. Scotti, Al_2_O_3_ decorated with polyhedral silsesquioxane units: An unconventional filler system for upgrading thermal conductivity and mechanical properties of rubber composites, *Compos. Sci. Technol.* **2023**, *236*, 109977.

[21] F. Tian, J. Cao, W. Ma, Enhanced thermal conductivity and rheological performance of epoxy and liquid crystal epoxy composites with filled Al_2_O_3_ compound, *Polym. Test* **2023**, *120*, 107940.

[22] M. Zhou, H. Bi, T. Lin, X. Lü, D. Wan, F. Huang, J. Lin, Heat transport enhancement of thermal energy storage material using graphene/ceramic composites, *Carbon* **2014**, *75*, 314.

[23] Z. Wang, M. Yang, Y. Cheng, J. Liu, B. Xiao, S. Chen, J. Huang, Q. Xie, G. Wu, H. Wu, Dielectric properties and thermal conductivity of epoxy composites using quantum-sized silver decorated core/shell structured alumina/polydopamine, *Compos. Part A-Appl. Sci. Manuf.* **2019**, *118*, 302.

[24] J. N. Song, Z. L. Peng, Y. Zhang, Enhancement of thermal conductivity and mechanical properties of silicone rubber composites by using acrylate grafted siloxane copolymers, *Chem. Eng. J.* **2020**, *391*, 123476.
